# Profiling of secondary metabolites and DNA typing of three different *Annona* cultivars grown in Egypt

**DOI:** 10.1007/s11306-022-01911-w

**Published:** 2022-07-04

**Authors:** Mona Arafa Mohammed, Manal A. Hamed, Souad Eisawy El-Gengaihi, Ahmed Mahmoud Aboul Enein, Piotr Kachlicki, Emad Mohamed Hassan

**Affiliations:** 1 Medicinal and Aromatic Plants Research Department, Pharmaceutical and Drug Industries Research Institute, National Research Centre, Dokki, Cairo, 12311 Egypt; 2 Department of Therapeutic Chemistry, Pharmaceutical and Drug Industries Research Institute, National Research Centre, Dokki, Cairo, 12311 Egypt; 3 Biochemistry Department, Faculty of Agriculture, Cairo University, Giza, Egypt; 4 Institute of Plant Genetics of the Polish Academy of Sciences (Metabolomics Group), Poznan, Poland

**Keywords:** Annona Abdel Razek, Gastric ulcer, antioxidants, Enzymes, Polyphenolics, Alkaloids

## Abstract

**Objectives:**

Natural products are often efficacious and safe alternatives to synthetic drugs. This study explored secondary leaves and bark metabolites profiles in extracts of a new Egyptian hybrid, *Annona cherimola* × *Annona squamosa*, known as Abdel Razek. This hybrid exhibited 100% similarity with *A. cherimola* as evidenced by random amplified polymorphic DNA (RAPD) and inter-simple sequence repeat (ISSR) analyses.

**Methods:**

Primary constituents in methanol extracts of different plant organs were identified. Extracts richest in alkaloids and polyphenolics were assessed for in vitro antioxidant activity and the most potent were further studied in vivo for treating gastric ulcer in rats. The latter activity was assessed histopathologically.

**Results:**

Structural analysis with HPLC/ESI-MS^n^, and UPLC/HESI-MS/MS identified 63 metabolites, including seven amino acids, 20 alkaloids, 16 flavonoids, eight phenolics and other compounds. Severe stomach alteration was observed after ethanol induction in rats. Ulcer score, oxidative stress biomarkers, cell organelles biomarker enzymes, and gastrointestinal histological features improved to variable degrees after treatment with *Annona* Abdel Razek hybrid leaves and bark methanol extracts.

**Conclusion:**

Extracts of *Annona* Abdel Razek had showed in vitro antioxidant effect and may be promising for the treatment of gastric ulcers.

**Graphical abstract:**

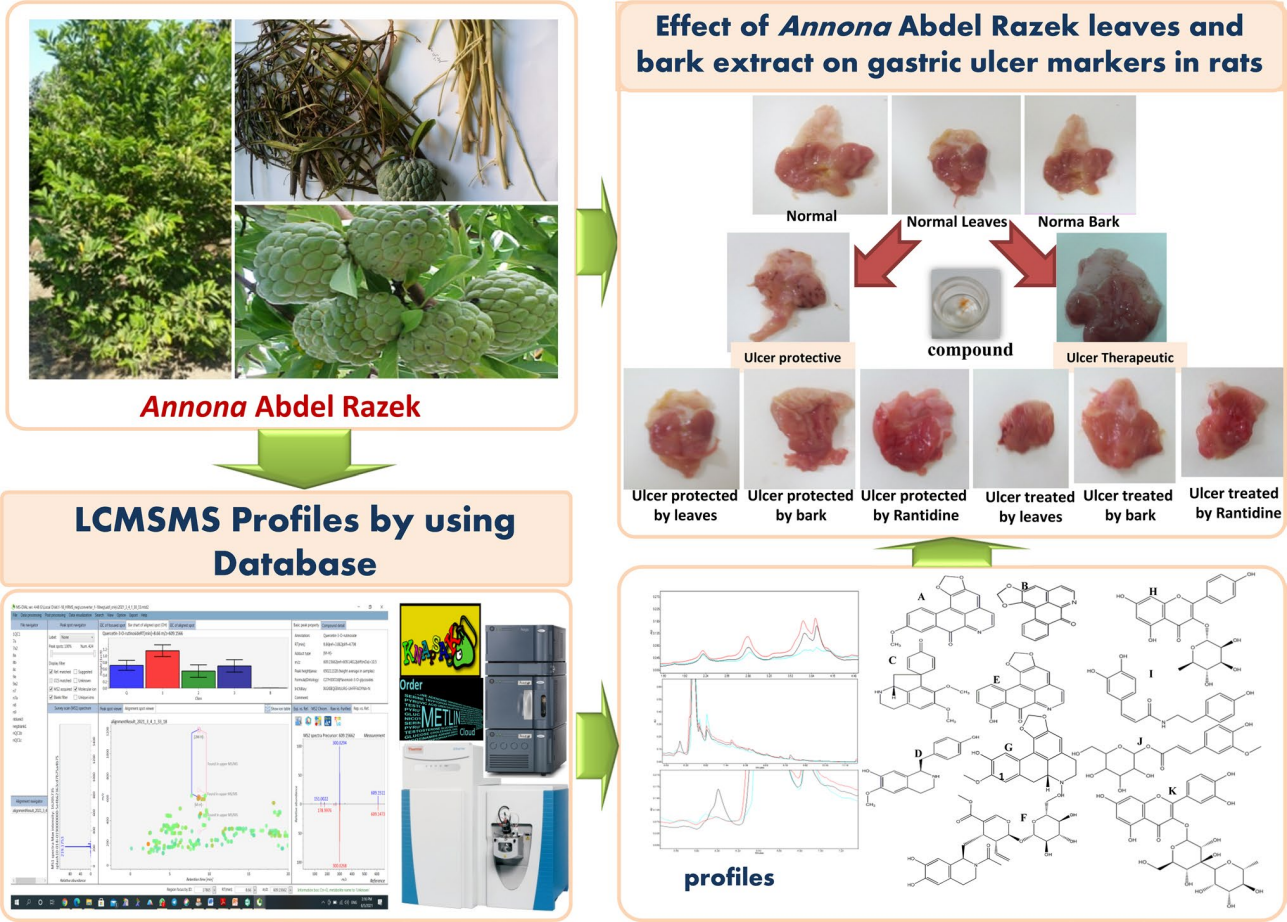

**Supplementary Information:**

The online version contains supplementary material available at 10.1007/s11306-022-01911-w.

## Introduction

Gastric ulcers affect a large percentage of world's population and are caused by a variety of conditions, including stress, smoking, alcohol intake, nutritional deficiencies, *H. pylori* infection, and NSAIDs (Martel et al., 2020). A metabolic imbalance in stomach mucosal defenses is involved in the pathogenesis of ulceration. Acid, pepsin, and *H. pylori* are common stressors, and mucus, prostaglandins, bicarbonate, nitric oxide, and growth factors are protective elements (Graham, 2014). Damage to the gastrointestinal mucosa caused by alcohol involves microvascular injury, rupture of the vascular endothelium, increased vascular permeability, edema, and epithelial elevation (Penninger et al., 2020). Treatment of peptic ulcers first focuses on lowering stomach acid production, and then on restoring gastric mucosal protection. Proton pump inhibitors, histamine receptor blockers, mucosal barrier medicines, and prostaglandin analogues are all used in modern treatment (Graham, 2014).

Several species of *Annona* (Family: Annonaceae) are used in traditional medicine, reflecting their anti-anxiety, antidepressant, anticonvulsant, and tranquilizing properties (Martínez-Vázquez et al., 2012). *Annona* is the second largest genus in the family, containing approximately 166 species (Nagy & Wardowski, 1990). *A. squamosa* and *A. cherimola* and their hybrid (Abdel Razek) rich produce substantial amounts of secondary metabolites, including phenolics, flavonoids, alkaloid isoquinolines, peptides, acetogenins, lectins, and volatile oils (Dahiya & Dahiya, 2021). Acetogenins are most commonly isolated from *A. muricata*, *A. squamosa*, *A. cherimola* and *A. classiflora* (Prabhakaran et al., 2016). This situation prompted scientists to look for novel anti-ulcer medications in natural materials due to the fact that the side-effects of the natural compounds are comparatively lower than those of the synthetic ones. Therefore, many *Annona* species exhibit anticancer, antioxidant, antibacterial, and antifungal properties (Leiteet al., 2020). Such properties are the basis for substantial research on natural products (Huang et al., 2019), and the present study explored secondary metabolites in leaves and bark from the new hybrid *Annona* Abdel Razek for their antioxidant and anti-ulcerative effects in rats.

## Material and methods

### Phytochemical study

#### Chemicals

Analytical grade solvents and chemicals used in this study were purchased from Fisher Scientific Co. (Bishop Meadow Road, Loughborough, UK).

#### Plant products and DNA fingerprinting

Tissues from three *Annona* cultivars, *A. squamosa*, *A. cherimola,* and Abdel Razek, were collected from a private farm in Mansoriya district, Giza, Egypt. Plants were identified by Prof. Dr Mona Marzouk, Plant and Systematic Department at the National Research Centre, Egypt and voucher specimens numbers 521, 522, and 523 were deposited in the herbarium. Presence/absence of RAPD and ISSR markers were scored using DNA fragments of gels (Tables 1, S1–S3). Only five primers were successful in producing repeatable polymorphic DNA products. A UV transilluminator was used to record RAPD and ISSR patterns (Pharmawati et al., 2004).

**Table 1:** RAPD and ISSR primers and nucleotide sequences

	Nucleotide sequences for RAPD-PCR Analysis		Nucleotide sequences for ISSR procedure	
No	Name	Sequence	Name	Sequence
1	OP- A01	5′ GAA AGG GGT G 3′	14A	5′ CTC TCT CTC TCT CTC TTG3′
2	OP-B02	5′ TCG GGG ATA G 3′	44B	5′ CTC TCT CTC TCT CTC TTG3′
3	OP-B07	5′ GAA AGG GGT G 3′	HB-08	5′ GAG AGA GAG AGA GG3′
4	OP-B11	5′ GTA GAC CCG T 3′	HB-11	5′ GTG TGT GTG TGT TGT CC 3′
5	OP-C04	5′ CCG CAT CTA C 3′	HB-15	5′ GTG GTG GTG GC 3′

#### Successive extraction and evaluation of total polyphenolics, flavonoids, and alkaloids *A.* Abdel Razek plant tissues

Five hundred grams from different parts (leaves, barks, fruits, and seeds) were extracted by soaking in 10 L MeOH (100, 80, and 50%) for 48 h at room temperature. Using a rotary evaporator, the mixed alcoholic extracts were concentrated under decreased pressure at 40 °C. The complete extract of each part was suspended in 0.5 L water for 24 h, then partitioned with 0.5 L of different solvent (Me_2_Cl_2_, EtOAc), and BuOH before being filtered through Whatman No. 54. Total flavonoids and polyphenols were assessed according to (Hamed et al., 2019), whereas total alkaloids were determined according to (El-Gengaihi et al., 2020).

#### Profiling of secondary metabolites using LC–MS/MS

Two hundred mg of dried leaves and bark were separately sonicated with two ml methanol for 5 min at 37 °C. The filtered extract was injected in negative and positive ion mode into an ion-trap Esquire 3000 mass spectrometer with an ESI ion source. Analysis conditions for nebulizer, dry gas flow, and dry gas temperature were 30 psi, 9.0 L/min, and 310 °C, respectively. Skimmer 1 was set to a negative voltage of − 10.0 V. Scan resolution was 13,000 m/z/sec with a range of 150–3000 m/z. An Acquity system (Waters, Milford, USA) was combined with a Q-Exactive hybrid MS/MS quadrupole—Orbitrap mass spectrometer (Thermo, Bremen, Germany). A BEH shield C18 column (1502.1 mm, 1.7 m) was used for chromatographic separation with deionized water acidified with 0.1 percent formic acid (solvent A) and acetonitrile (solvent B). The mobile phase gradient was 0–14 min from 5% B to 50% B, then 14–20 min to 98% B, and sustained for 5 min. The system was reset to initial settings and re-equilibrated for 3 min between runs. Q-Exactive MS conditions were HESI ion source voltage of − 3 kV or 3 kV, gas (N2) flow rate of 48 L/min, and auxiliary gas flow rate of 13 L/min. Ion source capillary and auxiliary gas heater temperatures were 250 °C and are 380 °C, respectively. Collisions power for CID MS/MS studies was 15 eV (Mohammed et al., 2021).

#### Data processing

MSMS fragmentation patterns were generated for 63 phenolics, alkaloids, terpenes, and other metabolite using ion mobility tandem mass spectrometry to allow identification of extract composition. These four fractions from raw MS data were output to analysis base file (abf) format and packaged in MS-DIAL 4.60 (http://prime.psc.riken.jp/). MSP format libraries were used with enhanced standardized untargeted lipidomic and metabolomic patterns to filter noisy spectra via classical spectral similarity calculations. Selected metabolites were identified by comparing fragmentation patterns and retention indices with similar information from KNApSAcK, Metlin, Golm, MassBank, Fiehn Bin Base, LipidBlast, ReSpect, and RIKEN databases (Ammar et al., 2021; Mohammed et al., 2021).

### Pharmacological Investigation

#### In vitro antioxidant analysis

The ABTS^+^ was determined in various extracts using the methods provided by (Dinkova-Kostova et al., 2007)). The DPPH (1,1-diphenyl-2-picrylhydrazyl) free radicals scavenging test was carried out according to Ammar et al. (2021). The Percent of inhibition of DPPH and ABTS^+^ radicals was estimated using the following formula:

$${\text{Percent}}\,{\text{of}}\,{\text{inhibition}} = [({\text{A}}_{{{\text{control}}}} - {\text{A}}_{{{\text{sample}}}} )/{\text{A}}_{{{\text{control}}}} ] \times 100,$$

where A is the absorbance of DPPH^−^ and ABTS^+^ at 517 and 734 nm, respectively.

#### Acute toxicity

Forty-eight rats, weighing 100–120 g were separated into two groups of 24 animals. Each group was subdivided into three subgroups of eight. Each animal received an oral dose of 250, 500, or 1000 mg/kg body weight of Abdel Razek leaves or bark alcoholic extract. Numbers of dead animals were counted over 15 days. The mortality rate and LC50 were also recorded. Extracts were safe at all tested concentrations, and a dose of 500 mg/kg body weight was chosen for further investigation (El-Gengaihi et al., 2013).

#### Doses and administration routes

Rats were administered 0.5 ml/100 g body weight absolute ethanol orally after a 24-h fast (Fahmi et al., 2019). Abdel Razek extracts were administered orally for 1 week after ulcer development in therapeutic model and 1 week before ulcer development as protective model (Mohammeda et al., 2020). Ranitidine given orally (100 mg/kg bw/day for one week) as a reference drugs for antiulcer effects (Fahmi et al., 2019).

#### Experimental groups

Seventy-two male albino Wistar rats were divided equally into nine groups.

Group 1 rats served as controls.

Groups 2and 3 animals received alcoholic extracts of bark or leaves daily for 1 week.

Group 4 rats received ethanol dose on an empty stomach for 24 h. Animals were euthanized one hour later to provide a baseline for ulcer production.

Groups (protective model) 5 and 6 rats received plant extracts or ranitidine daily for 7 days before receiving one oral dose of absolute ethanol on an empty stomach for 24 h and sacrificed 1 h later.

Group 7 animals received ethanol and then euthanized after seven days.

Groups (therapeutic model) 8 and 9 rats received one oral dose of absolute ethanol and then plant extracts or ranitidine daily for 7 days and sacrificed 24 h after the last administration.

#### Samples preparations

Stomach tissue was homogenized in a normal physiological saline solution (0.9% NaCl) (1:5 w/v). The homogenate was centrifuged for 15 min at 300 g at 4 °C, and the supernatant kept at − 80 °C until further analysis (Khalaf-Allah et al., 2016).

#### Ulcer index

##### Stomach lesion counts

Stomachs were removed, opened from the long curvature, cleaned with saline solution, expanded, and placed on the dissection plate. Numbers of lesions were counted using a magnifying glass (Guedes et al., 2008; Mohammeda et al., 2020).

##### Stomach total acidity

Gastric contents were collected and centrifuged for 15 min at 300* g*. Supernatant volumes (ml) were measured and total acidity determined by titration with 0.1 N NaOH using 2% phenolphthalein as an indicator. The results are expressed as milliequivalents per liter (mEq/L), where:

$${\text{mEq = Vol of NaOH}} \times {\text{normality of NaOH}} \times {\text{equivalent weight of HCl}} \times {\text{1000/Sample volume}}$$

#### Oxidative stress and cell organelles markers

Glutathione (GSH) (Bhardwaj et al., 2000), malondialdehyde (MDA) (Buege & Aust, 1978), superoxide dismutase (SOD) (Nishikimi et al., 1972), nitric oxide (NO) (Montgomery & Dymock 1961), catalase (CAT) (Shelton & Rice 1957), succinate dehydrogenase (SDH) (Swanson, 1955), lactate dehydrogenase (LDH) (Wattiaux & De Duve, 1956), glucose-6 phosphatase (G-6-Pase) (Bodansky & Schwartz, 1963), acid phosphatase (AP) (Bodansky & Schwartz, 1963), alkaline phosphatase (ALP), 5′-nucleotidase (5′NT) and total protein (Bradford, 1976) were measured in stomach tissue.

#### Histopathological analysis

Slices of stomach tissues were embedded in paraffin blocks after fixation in 10% formalin. Masson’s trichrome and hematoxylin and eosin (H&E) were used to stain 5 µm thick sections. Sections were examined under a light microscope for pathological abnormalities (Bancroft, 1996).

#### Statistics

All biochemical data are presented as means ± SD of eight rats per group. Statistical analysis used one-way analysis of variance (ANOVA), with Costat Software, followed by least significance difference (LSD) tests between groups; p < 0.05 was used as a threshold for significance.

## Results

### DNA Fingerprinting

Primer OP-A01 showed eight bands of 270–1200 bp showing four monomorphic and four polymorphic bands indicating 50% polymorphism (Tables S3 and figure S1 A (supplementary data)). OP-B02 showed 11 bands of 260–1390 bp. Two monomorphic and nine polymorphic bands were noted, suggesting 81.82% polymorphism. Similar results for OP-B07, OP-B11, and OP-C04 were 11 bands of 155 to 1350 bp, 11 bands of 225 to 1455 bp, and seven bands of 180 to 680 bp, respectively. These primers displayed nine monomorphic and two polymorphic bands indicating 18.18% polymorphism, three monomorphic and eight polymorphic bands suggesting 72.73% polymorphism, and three monomorphic and four polymorphic bands indicating 57.14% polymorphism, respectively.

Likewise, primers A, 44B, HB-08, HB-11, and HB-15 produced six bands of 295–820 bp, eight bands of 280–640 bp, nine bands of 145–825 bp, 12 bands of 295–1040 bp, and eight bands of 340–1070 bp, respectively. These primers exhibited four monomorphic and two polymorphic bands suggesting 33.33% polymorphism, six monomorphic and two polymorphic bands indicating 25% polymorphism, no monomorphic and nine polymorphic bands suggesting 100% polymorphism, four monomorphic and eight polymorphic bands indicating 66.67% polymorphism, and five monomorphic and three polymorphic bands suggesting 37.5% polymorphism, respectively (figure *S1 B*).

### Phytochemical analysis

#### Total polyphenolic, flavonoid and alkaloid contents

Bark, fruit, and seed extracts showed lower total phenol and flavonoid content than leaves extracts (Table 2). The highest total polyphenolic and flavonoid concentrations were found in butanol and total alcohol extracts of Abdel Razek (18.36, 20.36, 3.2, and 4.2 mg/g, respectively). Further, total alkaloids extracted by different solvents were limited, and no significant differences were observed among plant parts extracts. The total alkaloid content was greater in bark than in seeds as 0.08 vs. 0.06 mg/g DW, respectively*.*

**Table 2:** Total polyphenolic, flavonoid and alkaloid contents

*Annona* Abdel Razek parts	Chloroform		Ethyl Acetate	Butanol	Total alcohol
Total phenolics (mg/g Dw)					
Leaves	1.3 ± 0.02	15.65 ± 0.36		18.36 ± 0.21	20.36 ± 0.32
Bark	0.9 ± 0.10	13.36 ± 0.42		15.36 ± 0.25	12.87 ± 0.46
Fruit	0.2 ± 0.01	8.96 ± 0.20		6.32 ± 0.42	5.65 ± 0.85
Seed	0.12 ± 0.01	6.65 ± 0.21		8.63 ± 0.52	7.89 ± 0.45
Total flavonoids (mg/g Dw)					
Leaves	0.07 ± 0.01	1.02 ± 0.02		3.2 ± 0.03	4.2 ± 0.01
Bark	0.04 ± 0.00	0.98 ± 0.21		1.86 ± 0.21	0.98 ± 0.02
Fruit	0.02 ± 0.00	0.78 ± 0.10		2.32 ± 0.02	1.9 ± 0.01
Seed	0.02 ± 0.00	0.45 ± 0.02		0.87 ± 0.14	0.6 ± 0.02
Total alkaloids (mg/g Dw)					
Leaves	0.01 ± 0.002	0.001 ± 0.005		0.009 ± 0.002	0.05 ± 0.005
Bark	0.02 ± 0.001	0.001 ± 0.004		0.02 ± 0.001	0.08 ± 0.004
Fruit	0.01 ± 0.006	0.001 ± 0.001		0.01 ± 0.001	0.05 ± 0.003

### Metabolomics analysis

**Fig. 1:**
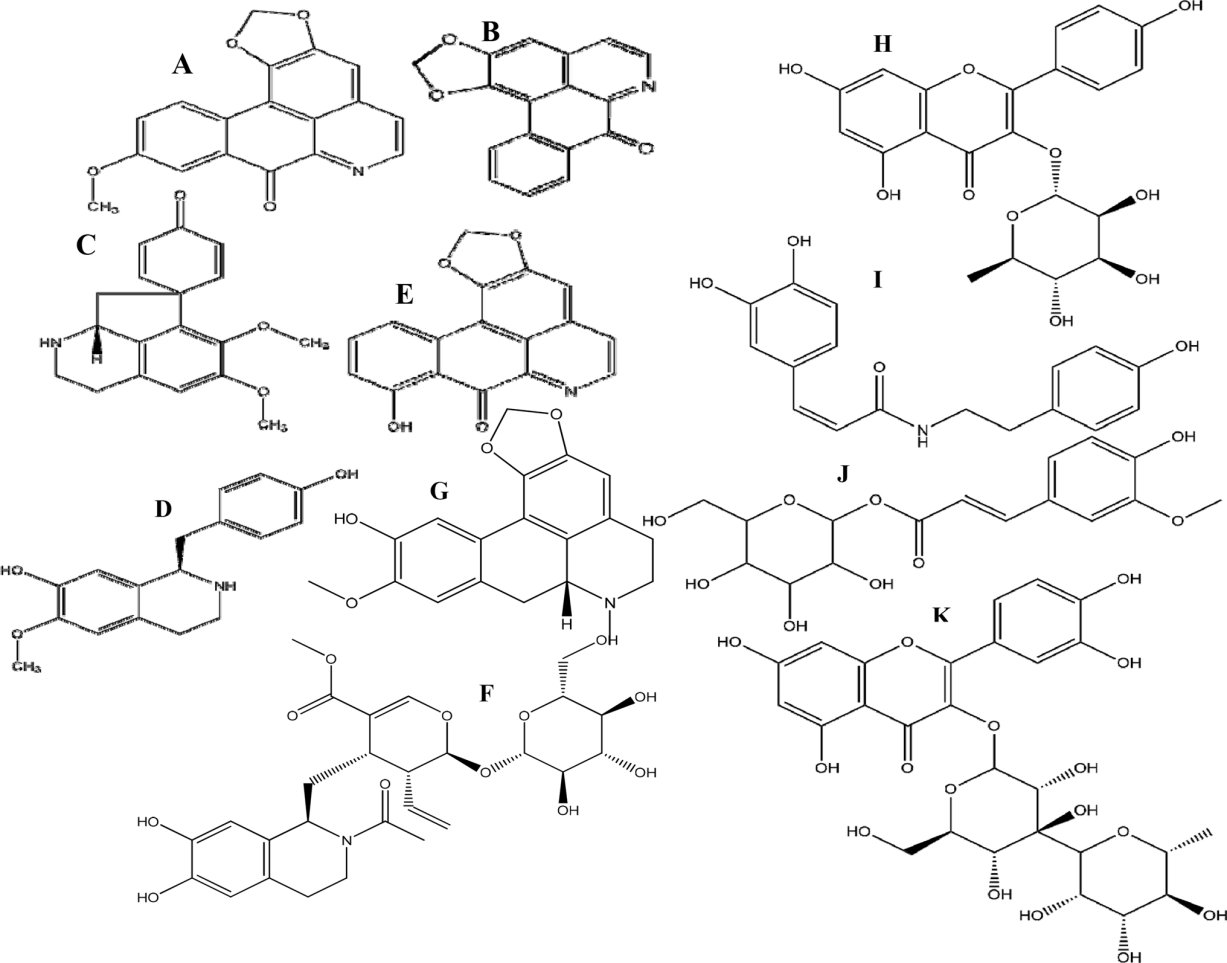
Structure of compounds **A** Lanuginosine, **B** Liriodenine, **C** Stepharine, **D** Coclaurine, **E** Oxostephanosine, **F** Ipecoside, **G** (−)-Pphanostenine, **H** Kaempferol-*O*-3-alpha-rhamnopyranoside, **I** N-cis-caffeoyltyramine, **J** Caffeic acid hexoside and **K** Rutin

**Fig. 2:**
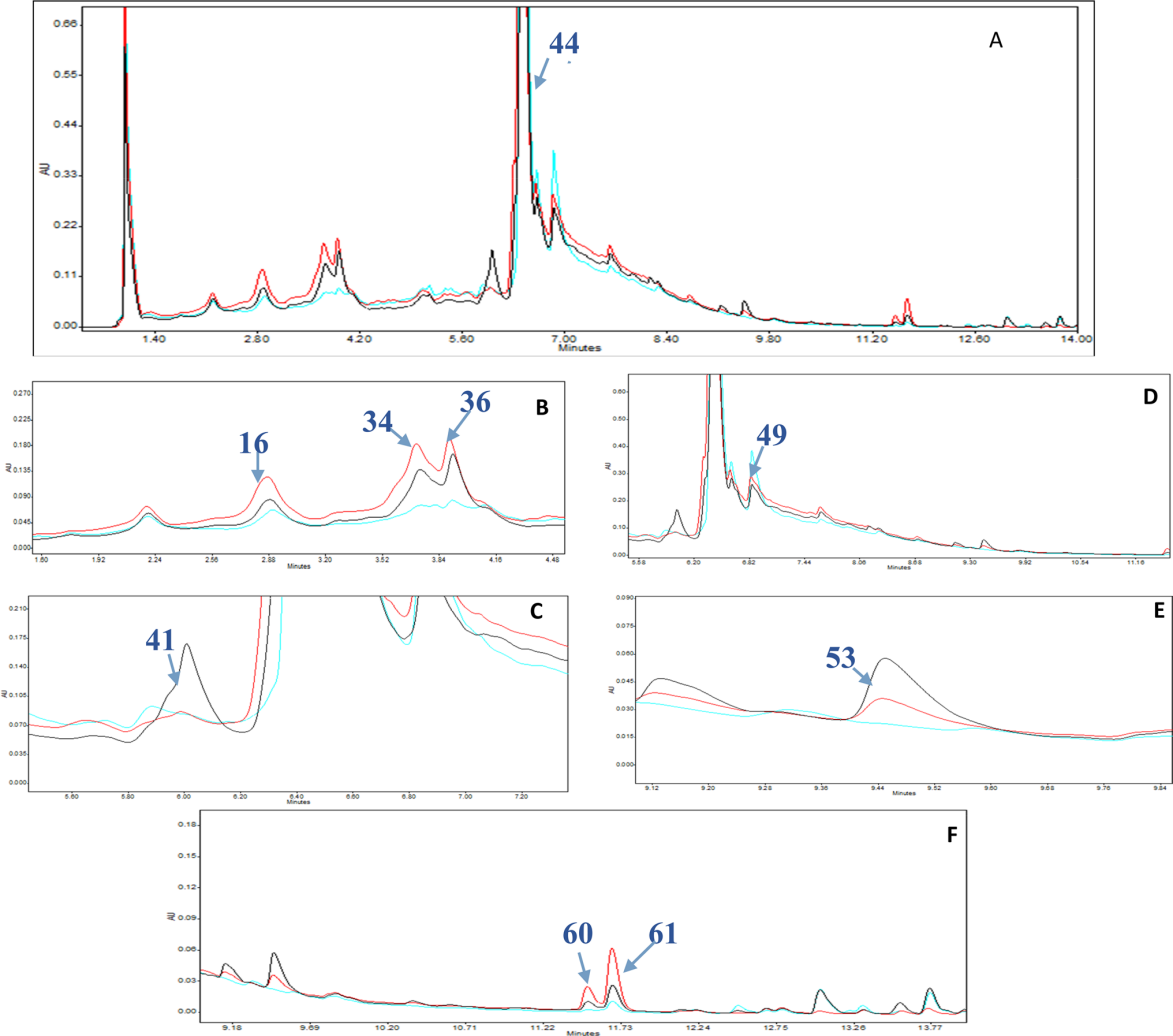
**A** Major metabolites by comparison profiles between UPLC-UV chromatograms of compounds recorded at 330 nm of *Annona* species, where *A. cherimola* (black line)*, A. squamosa* (green line) and *A.* Abdel Razek (red line).

Metabolomic studies established profiles of 63 compounds, consisting of phenols, flavonoids, coumarins, amino acids, and alkaloids, from leaves and bark. Compounds were identified by comparing their molecular mass (< 5 ppm), mass spectroscopy, and retention times to standard compounds, data from the ChEBI, PubChem, Metlin, and KNApSAck databases, and results from earlier research. Several compounds co-eluted from the LC column, and the mass spectrometer was unable to fragment these compounds independently. This limitation was addressed by: (i) extending the separation period of HPLC-MS^n^ runs to 1 h and (ii) using a C18 reversed-phase UPLC-HESI-MSMS to analyze fractions derived from polyamide preparative LC runs. Secondary metabolites included alkaloids, phenolic acids, flavonoids and polyphenols, such as coumaric acid, coumaroyl quinic acid, kaempferol, feruloyl hexoside, feruloyl-quinic acid, caffeoyl quinic acid, caffeic acid, caffeic acid hexoside, epicatechin, catechin, alkaloids (boldine, caranine, oxoanolobine, reticuline, ipecoside, coclaurine), and amino acid derivatives (n-fructosyl isoleucine, n-fructosyl pyroglutamate, and tryptophan) (Table 3 and Figs. 1, 2, & S3).

**Table 3:** Secondary metabolites profiles identified in *Annona* Abdel Razek leaves and bark using complementary systems: HPLC-ESI-MS^n^ and UPLC-HESI-MS/MS

No	Rt [Min]	Tentative metabolite identification	Classes	Chemical formula	HRMS of exact mass measured, calculated and fragmentation ions				∆ ppm	Fragentation in Low Resolution		λ max [nm]	Referencess
					[M–H]^−^	Fragmentation ions	[M + H]^+^	Fragmentation ions		Negative ion mode ESI (−)	Positive ion mode ESI (+)		
*Amino acids groups and organic acids*													
**1**	**1.72**	2,6-dihydroxy-4-Methoxytoluene^a,b^	Organic compound	C_8_H_10_O_3_	153.0546, 153.0546	123.0438, 109.0282	155.0703, 155.0703	113.9643, 109.1015	0.1376	–	–	237, 280	XCMS, MS-Dial
**2**	**2.25**	N-Fructosyl isoleucine^a^	Amino acid	C_12_H_23_NO_7_	292.1405, 292	150.0040, 130.0860	294.2052, 294.2100	276.1441, 230.1387, 132.1021	4.866/5.2520	–	–	208, 225	XCMS, MS-Dial
**3**	**2.48**	N-Fructosyl pyroglutamate^a,b^	Amino acid	C_11_H_17_NO_8_	290.0872, 290.0870	230.0672, 200.0559, 170.0447, 128.0340	–	–	0.7132	–	–	213, 222, 270	MS-Dial
**5**	**3.29**	Pantothenic acid^a^	vitamin	C_9_H_17_NO_5_	218.1027, 218.1023	146.0809, 88.0388	–	–	1.9399	–	–	–	MS-Dial
**10**	**3.88**	Tryptophan^a,b^	Amino Acid	C_11_H_12_O_2_N_2_	203.0818, 203.0815	142.0652, 116.0489, 97.0283	–	–	1.2432	–	–	215, 239, 258	MS-Dial
**27**	**5.61**	Acetylleucine^a,b^	Amino Acid	C_8_H_15_NO_3_	172.0969, 172.0968	130.0860	–	–	0.2497	–	–	–	MS-Dial
**28**	**5.72**	Methoxytyrosine^a,b^	Amino Acid	C_10_H_13_NO_4_	210.0792, 210.0743	124.0391, 94.0281	212.0918, 212.0917	194.0801, 145.0498, 127.0394, 97.0290	0.7146/0.1506	–	–	203, 218	MS-Dial
**35**	**6.31**	AcetylPhenylalanine^a,b^	Amino Acid	C_11_H_13_NO_3_	206.0218, 206.0812	164.0706, 147.0439, 91.0538, 72.0076	208.0970, 208.0968	190.1308, 151.0758, 104.0471	0.7931	–	–	237, 280, 306	MS-Dial
*Alkaloids groups*													
**4**	**3.10**	Boldine^b^	Aporphine Akaloids	C_19_H_21_NO_4_	326.1380, 326.1387	178.9966, 164.0706, 147.0441, 101.0230	328.1541, 238.1543	297.1125, 285.1139, 265.0860, 283.060, 237.0909, 178.0865, 121.0651, 58.0660	2.0040/− 0.5801	–	328, 283,, 249, 175, 121	213, 222, 258	(Wu et al., 1993)
**8**	**3.64**	Ipecoside^b^	Alkaloid terpene glycosides	C_27_H_35_O_12_N	564.2158, 564.2161	504.3117, 207.1137, 162.0549	566.2220, 566.2245	434.1812, 272.1281, 161.0598, 107.0.98	2.1690	564, 504, 207	566, 434, 273, 175, 272, 161, 107	221	(Harborne, 1999) KNApSAcK
**9**	**3.79**	Caranine^a,b^	Indolizidine Alkaloids	C_16_H_18_O_3_N	270.1140, 270.1144	162.0550	272.1281, 272.1281	255.1016, 161.0598, 123.0445, 107.0497	0.1384	–	272, 255, 107, 161, 123	250, 350	(Harborne, 1999) KNApSAcK
**12**	**4.20**	Fenfangjine G^b^	Isoquinolines, Alkaloid	C_22_H_28_NO_8_	432.1667, 432.1647	270.1138, 162.0549	434.1810, 434.1809	416.1701, 387.2764, 309.1223, 255.1021, 174.0759, 107.0495	0.1302	–	434, 272, 161, 255, 107	206, 234, 250, 350	(Ogino et al., 1998) KNApSAcK
**13**	**4.50**	Oxoanolobine^a,b^	Oxoaporphine Alkaloids	C_17_H_20_O_4_N	–	–	302.1387, 302.1392	285.1118, 270.0853, 253.0851, 191.0703, 107.0496	− 1.8151	–	302, 285, 153, 107, 253, 191, 107	234, 310	(Simeon et al., 1989) KNApSAcK
**14**	**4.53**	(-)-Phanostenine^a,b^	Aporphine alkaloid	C_19_H_20_O_4_N	–	–	326.1386, 326.1387	295.0967, 265.0859	− 3.9663	–	326, 297, 218, 151, 158	–	(Rabêlo et al., 2013) KNApSAcK
**16**	**4.82**	Tembetarine^a,b^	Isoquinolines, Alkaloid	C_20_ H_26_O_4_N	–	–	344.1852, 344.1856	299.1281, 165.0548, 147.0441	− 1.1421	–	344, 299,165, 279, 192, 177	230, 280	(Nishiyama et al., 2004) KNApSAcK
**18**	**5.23**	Corytuberine ^a,b^	Aporphine Alkaloid	C_19_H_21_NO_4_	–	–	328.1553, 328.1557	297.1129, 265.0859, 237.0912, 178.064	-1.0290	–	328, 297, 178, 265, 237, 191	203, 277, 300	(Harborne, 1999) KNApSAcK
**19**	**5.28**	Laurifoline^a,b^	Aporphine Alkaloid	C_20_H_24_NO_4_^+^	340.1558, 340.1557	325.1324, 310.1091, 179.0340, 135.0441	342.1697, 342.1700	297.1123, 265.0859, 237.0908, 178.0867, 143.9961	-0.7188	–	342, 297, 256, 245	270	(Saxena, 1979) KNApSAcK
**22**	**5.45**	Reticuline^a,b^	Benzylisoquinoline Alkaloids	C_19_H_23_O_4_N	328.0965, 328.0968	191.0553	330.1693, 330.1686	299.1274, 192.1020, 175.0755, 137.0597	2.0276	–	330, 299, 192, 175	220, 280	(Simeon, Rios, Villar 1989) KNApSAcK
**29**	**5.80**	Coclaurine ^b^	Isoquinoline Alkaloids	C_17_H_18_NO_3_	284.1258, 284.1300	245.0671, 176.0707, 107.0488	286.1440, 286.1438	269.1173, 237.0916, 175.0967, 143.0494, 107.0496	1.9256	–	286, 269, 107, 237, 175, 137, 107	280, 320	(Nishiyama et al., 2006)
**30**	**5.84**	Anomoline^b^	Isoquinoline alkaloids	C_18_H_22_O_4_N	314.1398, 314.1400	180.0652, 121.0280	316.1543, 316.1543	299.1279, 267.1009, 121.0651	3.6460/0.2160	–	316, 299, 267, 205, 107		(Harborne, 1999)
**33**	**6.15**	Thalifoline ^b^	Quinolone Alkaloids	C_11_H_12_O_3_N	206.0819, 206.0812	164.0707, 147.0439	208.0970, 208.0968	190.1308, 151.0758, 104.0471	3.6620	206, 164, 147		211, 278	(Chen et al., 1999)
**39**	**7.18**	Dehydronuciferine	Isoquinoline Alkaloids	C_19_H_19_NO_2_	–	–	294.1483, 294.1489	265.2290, 249.0909, 147.0441	–	–	–	–	(Liu et al., 2014) metlin
**52**	**9.80**	Liriodenine ^b^	Aporphine Alkaloids	C_17_H_9_ O_3_N	–	–	276.0656, 276.0655	188.0970	0.0668		276,188, 117, 75	247, 269, 302	(Chen & Wu, 2001)
**53**	**9.90**	Oxostephanosine ^b^	Aporphine Alkaloids	C_17_H_9_O_4_N	–	–	292.0604, 292.0604	237.0477, 177.0917	− 0.1370	–	292, 263, 234, 205, 177, 150	215, 245, 275, 320, 364,448	(Pharadai et al., 1985)
**54**	**10.30**	Lanuginosine^a,b^	Aporphine Alkaloids	C_18_ H_11_O_4_N			306.0761, 306.0761	291.0513, 263.0513, 222.0036, 179.0766	0.0865		306, 275, 263, 221, 205	246, 271, 315	(Chen et al., 1998)
**60**	**12.47**	( +)-Stepharine^b^	Proaporphine Alkaloids	C_18_H_19_O_3_N	296.1287, 296.1281	–	298.1440, 298.1438	177.0547, 145.0284	− 1.8519/0.6085	296, 251, 183	298, 177, 145, 117	279	(Chen et al., 1997)
**61**	**12.58**	N-Methylcrotsparine^b^	Proaporphine Alkaloids	C_18_ H_20_O_3_N	296.1294, 296.1300	281.1060, 190.0509	298.1436, 298.1438	280.2633, 250.2538, 177.0546, 163.0390, 105.0705	− 2.0297/− 0.7222	–	298, 269, 192, 161, 107	290, 350	(Simeon et al., 1989) KNApSAcK
**62**	**12.70**	Crabbine^b^	Aporphine Alkaloids	C_20_H_24_O_5_N	–-	–	358.1654, 358.1642	313.1433, 281.1169, 135.0807	− 3.6119	–	358, 313, 281, 190, 135	212, 280	(Yang et al., 1993)
*Polyphenol and Flavonoid Groups*													
**6**	**3.38**	Catechols^a,b^	Organic compound	C_6_H_6_O_2_	109.0280, 109.0284	81.0331, 67.0173	–	–	− 3.5845	–	–	–	MS-Dial
**7**	**3.58**	N-cis-caffeoyltyramine^a,b^	Phenolic acid	C_17_H_17_O_4_N	–	–	300.1233, 300.1230	251.1083, 175.0755, 121.0652, 107.0498	0.7529		300, 107	215, 234, 280	(Chen et al., 1998)
**11**	**4.17**	b-D-glucoside of 1-methyl iso lariciresinol^b^	Lignan Glycosides	C_27_H_35_O_12_	548.1784, 548.1790	254.0822	550.1905, 550.1898	256.0295	1.3099	548, 254, 238	550, 256, 184	280, 330	(Martins & Nunez, 2015)
**15**	**4.60**	Benzoic acid + 1O, 2MeO, O-Hex^b^	Hydrolyzable tannins	C_15_H_20_O_10_	359.0964, 359.0973	197.0450, 182.0212, 153.0547	–	–	− 2.4176	–	–	201, 222, 270	MS-Dial
**17**	**5.20**	Coumaroyl quinic acid	Phenolic acid	C_16_H_18_O_8_	337.0782, 337.0765	191.0555, 163.0385, 119.0486	–	–	5.0711	–	–	234, 275	MS-Dial
**20**	**5.36**	(Afzelin) Kaempferol 3-O-alpha-rhamnoside^a^	Flavonoid	C_21_ H_20_ O_10_	431.0973, 431.0978	282.0511, 89.0226	–	–	− 1.2721	431, 281, 235, 178, 131, 89	433, 476, 415, 361, 272, 361, 272, 184, 123, 363, 294, 251, 174, 209, 110	270	(Chang et al., 1998a) KNApSAcK
**21**	**5.42**	6,7-Dihydroxycoumarin^a,b^	Coumarins	C_9_H_6_O_4_	177.0184, 177.0182	133.0282, 105.0328	179.0349, 179.0339	151.0388, 123.0444, 109.0652	0.8569 /0.7925	-	-	206, 237, 280	MS-Dial
**23**	**5.52**	Feruloyl Hexoside^a,b^	Phenolic acid	C_16_H_20_O_9_	355.1042, 355.1024	193.0499, 149.0595, 103.0022, 85.0279	–	–	5.2350	355, 191, 129, 85	–	210, 239	MS-Dial
**24**	**5.55**	Caffeoyl quinic acid^a,b^	Phenolic acid	C_16_H_18_O_9_	353.0883, 353.0881	191.0554, 179.0340, 161.0236, 135.0437, 85.0281	355.1023, 355.1025	163.0390, 145.0288, 117.0288	0.1650	353, 323, 191,135	–	213, 232, 280	(Simeon et al., 1989)
**25**	**5.57**	Caffeic acid hexoside^a^	Phenolic acid	C_15_H_18_O_9_	341.0793	191.0552, 179.0341, 135.0439	343.0818, 343.0812	191.0338, 147.0441	–	–	–	213, 234, 277	MS-Dial
**26**	**5.68**	Coumaric acid	Phenolic acid	C_9_H_8_O_3_	163.0390, 163.0390	119.0489	165.0547, 165.0546	147.0442, 119.0493	0.0957	239, 277	–	–	MS-Dial
**31**	**5.86**	Feruloyl quinic acid^a,b^	Phenolic acid	C_17_H_20_O_9_	367.1038, 367.1024	193.0499, 173.045, 149.0233, 134.0360	–	–	3.9892	–	–	201, 234, 277	MS-Dial
**32**	**5.88**	Caffeic acid^a,b^	Phenolic acid	C_9_H_8_O_4_	179.0341, 179.0339	135.0438	181.0493, 181.0495	171.0653, 163.0390, 145.0283, 121.0652, 107.0860	0.9630/− 1.4617	–	–	208, 237, 277	MS-Dial
**34**	**6.16**	Catechin^a^	Flavanols	C_15_H_14_O_6_	289.0717, 289.0707	245.0814, 203.0708, 151.0389, 125.0230	291.0889, 291.0863	207.0562, 147.0440, 139.0392, 123.0444	3.5327	–	–	237, 289	MS-Dial
**36**	**6.34**	(−)-Epicatechin^a^	Flavanols	C_15_H_14_O_6_	289.0718, 289.0707	245.0816, 205.0500, 179.0340, 151.0390, 125.0229	–	207.0566, 179.0704, 147.0439, 139.0392, 123.0443	3.9550	–	–	237, 289	MS-Dial
**37**	**7.00**	Kaempferol 3-rutinoside-7-glucoside^a,b^	Flavonoid	C_33_ H_39_ O_20_	755.2029, 755.2035	285.0403,	–	–	− 0.7262	755, 593, 285, 230, 211		218, 282, 321	(Beckmann & Geiger, 1963) KNApSAcK
**38**	**7.10**	Isorhamnetin 3-glucoside^a,b^	Flavonoid	C_22_H_21_O_12_	477.1028, 477.1033	314.0437, 271.0280	–	–	− 1.1494	477, 314, 135	479, 460, 306	212, 321	(Santos & Salatino, 2000)
**40**	**7.69**	Quercetin 3-rutinoside-7-glucoside ^a,b^	Flavonoid	C_33_H_40_O_21_	771.1998, 771.1984	609.1608, 300.0278, 271.0238, 178.9975	–	–	1.8754	771, 609, 300, 271, 179	–	206, 284	(Beckmann & Geiger, 1963) KNApSAcK
**41**	**7.76**	Quercetin 3,7-O-beta-diglucopyranoside^a,b^	Flavonoid	C_27_H_29_O_17_	625.1399, 625.1405	301.0358	627.1555,627.1563,	303.0500, 314.1380, 177.0548	− 0.8772/1.1548	625, 579, 372, 301	627, 314, 177	222, 331	(Santos & Salatino, 2000) KNApSAcK
**42**	**8.09**	Kaempferol 3-rhamnosyl-(1- > 2)-galactoside ^a,b^	Flavonoid	C _27_ H_30_ O_15_	593.1523, 593.1501	285.0403	595.1642, 595.1663	475.4135, 287.0548	3.7887/− 3.4433	593, 285, 255	595, 558, 474, 335, 287, 236	226, 278	(Yasukawa & Takido, 1987)
**43**	**8.20**	Kaempferol 3-rhamnosyl-(1- > 2)-galactoside-7-glucoside^a,b^	Flavonoid	C_33_H_40_O_20_	755.2029, 755.2091	285.0403, 253.0823	757.2211, 757.2219	527.0624, 353.0652, 287.0549	1.0564	755, 594, 498, 425, 359, 299, 285	–	–	(Harborne & Baxter, 1999)
**44**	**8.49**	Rutin (Quercetin 3-O-rutinoside) ^a^	Flavonoid	C_27_H_30_O_16_	609.1584, 609.1814	300.0276, 178.9971	611.1592, 611.1607	303.0500	− 2.3241	609, 301, 178, 254	611, 465, 367, 303, 249, 272, 202, 153, 110	256, 352	(Mohammed, Hamed et al., 2021)
**45**	**8.50**	Hirsutrin (Quercetin 3-O-beta-d-glucopyranoside)	Favonoid	C_21_H_21_O_12_	463.0898, 463.0871	300.0277, 151.0025	465.1022, 465.1028	303.0497,	5.721/− 1.2461	463, 301, 255, 151	465, 303, 165, 229	223, 278	(Mohammed, Hamed et al., 2021)
**46**	**9.06**	Wogonin ^a^	Methoxyflavone	C_16_H_12_O_5_	283.0632, 283.0596	239.0709, 145.0281, 117.0330, 93.0329, 65.0233	–	–	–	–	–	–	(Santos & Salatino, 2000)
**47**	**9.30**	Trifolin (Kaempferol 3-O-beta-d-galactopyranoside) ^a^	Flavonoid	C_21_H_21_O_11_	–	––	449.1078, 449.1084	287.0551, 127.0394	− 1.2211		449, 287, 172	280	(Santos & Salatino, 2000) KNApSAcK
**48**	**9.50**	Plantaginin ^b^	Glycosyloxyflavone	C_21_H_20_O_11_	447.0942, 447.0922	284.0329, 255.0297, 161.0701	449.1081, 449.1090	303.0500, 287.0553, 216.1577	4.4498/ 0.5840	447, 284,, 383, 297, 221, 163, 123	449,303, 287, 279, 472	241, 277	(Santos & Salatino, 2000) KNApSAcK
**49**	**9.52**	Isorhamnetin 3-O-glucoside ^a^	Flavonoid	C_22_ H_22_ O_12_	477.1028, 477..1028	314.0438, 269.1769, 163.0378	479.1184, 479.119	317.0656, 293.0294	0.0645/− 1.1446	–	479, 295, 241, 166, 259, 163, 115, 97		(Santos & Salatino, 2000) KNApSAcK
**50**	**9.67**	Quercetin-3-O-vicianoside ^a,b^	Flavonoid	C_26_H_28_O_16_	595.1369, 595.1372	300.0276	597.1461 597.1460	303.0500, 97.0921	0.5040	595, 300, 135, 177		239, 270, 308	(Santos & Salatino, 2000)
**51**	**9.79**	Quercetin 3-sophorotrioside	Flavonoid	C_33_ H_39_O_22_	787.1927, 787.1927	478.0904, 385.0577, 352.0215	–	–	0.0000	787, 727, 661, 353, 385, 283, 216	789, 481, 303, 165	278, 280	(Harborne, 1999)
**55**	**10.42**	Cis N-Feruloyltyramine^a,b^	Phenolic Acid	C_18_H_19_NO_4_	312.12415, 312.1225	190.0501, 178.0500, 148.0517, 135.0441	314.1385, 314.1387	206.0819, 177.0546, 145.0284, 121.0652	− 0.6234/3.3380	312, 177, 135	314, 206, 177, 255	280, 360	(Chang et al., 1998b)
**56**	**11.65**	Quercetin^a,b^	Flavonoid	C_15_H_9_O_7_	301.0348, 301.0354	178.9983, 151.0026, 121.0281	303.2312, 303.2319	257.2276	1.8144/–2 .1619	301, 153, 149, 165, 137	303, 179, 121, 151, 273, 257, 229	215, 278, 241, 280	(Mohammeda et al., 2020)
**57**	**11.73**	Kaempferol^a,b^	Flavonoid	C_15_H_10_O_6_	285.0399, 285.0405	151.0033, 133.0291	-	–	− 1.9120		287, 153, 133, 165, 121		(Mohammed et al., 2020)
**58**	**12.03**	2'-Hydroxyflavanone^a,b^	Flavanone	C_15_H_12_O_3_	239.0713, 239.0703	145.0283, 119.0489, 93.0328	–	–	4.3872	239, 145, 119	241, 223, 129	241, 270	Ms-dial
**59**	**12.15**	Patrinoside ^b^	Iridoid Glucosides	C_21_H_34_O_11_	461.2014, 461.2017	321.2769, 210.0682	–	–	3.2736	461, 323, 143		212, 306	(Demirezer et al., 1999)
**63**	**15.10**	Apigenin^a^	Flavonoid	C_15_H_10_O_5_	269.0458, 269.0444	-	–	–	5.0438	269, 162	–	–	Ms-dial

Based on the basis of exact masses recorded in HESI-MS/MS, chemical formulae were estimated, and fragmentation paths were detailed using ESI-MS^n^

Superscript Letters represent tentative identification from total alcohol extract of Abdel Razek (a), leaves fraction (b), bark fraction

### Biochemical investigations

#### In vitro antioxidant effect

Evaluation of the in vitro antioxidant activity of the alcoholic extracts from leaves, bark, fruits and seeds of Abdel Razek hybrid, as well as the total alkaloids, flavonoids and polyphenolics fractions are presented in (Table 4). The leaves, fruits and bark of this species were examined against vitamin C and trolox as standards. The results revealed that both leaves and barks of hybrid Abdel Razek extract induced 16.66 and 13.33, 22.85 and 9.22% of inhibition by using DPPH^−^ and ABTS^+^ as free radicals scavenging agents_,_ respectively (Table 4).

**Table 4:** In vitro antioxidant effect of *Annona Abdel Razek* different plant parts

Sample Con. µg/ml	ABTS	DPPH
Vit. C	46.88 ± 0.15	52.67 ± 0.32
Trolox	31.72 ± 0.05	33.34 ± 0.06
Leaves extract	22.85 ± 0.10	16.66 ± 0.19
Barks extract	9.22 ± 0.25	13.33 ± 0.37
Fruits extract	117.50 ± 0.86	76.65 ± 0.48
Seeds extract	151.80 ± 0.85	101.4 ± 0.14
Total alkaloids extract	227.90 ± 0.45	94.11 ± 0.16
BuOH extract (flavonoids)	101.30 ± 0.1	47.5 ± 0.58
EtOAc extract (polyphenolics)	49.44 ± 0.07	50.82 ± 0.04

Data represented as % of inhibition of triplicate readings

#### In vivo protective effect of *Annona* Abdel Razek

##### Ulcer index

**Fig. 3:**
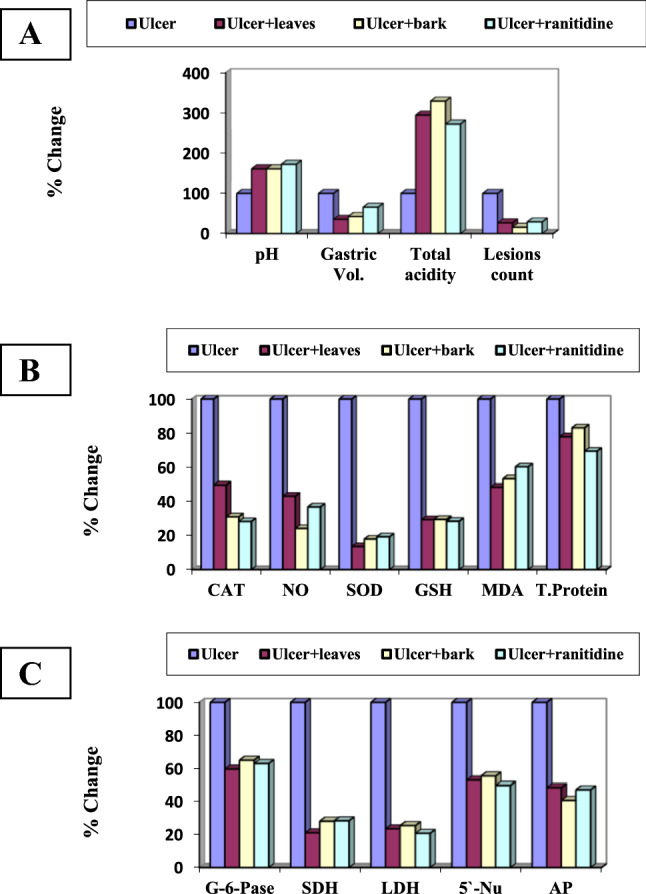
Percentage changes over ulcer group of the protective effect of *A.* Abdel Razek extracts and ranitidine on **a** gastric ulcer index, **b** antioxidant and protein levels and **c** cell organelles marker enzymes

Regarding the pH level in control rats protected with the plant extracts, the results revealed insignificant changes (Table S4 and Fig. 3a). Gastroulcerative rats recorded significant decrease by 40.90% compared with the control group. Gastroulcerative rats protected with the leaves and bark of *Annona* Abdel Razek, and ranitidine drug showed significant increase in the pH level by 61.53, 61.53, and 72.92%, respectively, compared with the ulcer group. The gastric volume in control rats protected with the plant extracts and ranitidine drug recorded insignificant changes indicating extract safety (Table *S4* and Fig. 3a). The ulcerative stomach showed significant increase in its volume content by 2260.08% compared with the control group. Protected rats with leaves and bark of *Annona* Abdel Razek and ranitidine drug recorded significant decrease in gastric volume by 64.40, 57.40 and 34.30%, respectively in comparison with the ulcer group. Regarding to the gastric total acidity, normal rats protected with the plant extracts showed insignificant changes as compared with the control group (Table *S4* and Fig. 3a). The gastric ulcerative rats showed significant decrease in the total acidity by 76.65% versus the control group. Protective rats with leaves and bark of the plant extract and ranitidine drug proved significant increase in the total acidity level by 194.00, 229.00 and 171.64%, respectively as compared with the ulcerative group. By examining the lesions counts in the stomach of ulcerative group, it showed eleven ulcer lesions per stomach (Table S4 and Fig. 3a). In protected rats with leaves and bark of the plant extract and ranitidine drug, the gastric lesions count decreased by73.33, 84.44 and 70.84%, respectively as compared with the ulcerative group.

##### Oxidative stress

The oxidative stress markers, protective rats with extracts of Abdel Razek and ranitidine drug produced did not significantly alter catalase, NO, SOD, glutathione, malondialdehyde and total protein levels as compared with control group (Table S5 and Fig. 3b). Significant increases in these indices were recorded in gastroulcerative rats amounted to 141.22, 280.00, 64.10 and 55.36%, respectively as compared with control group. Protection of gastro-ulcerative rats with leaves and bark Abdel Razek, and ranitidine drug recorded significant decrease in catalase activity by 50.11, 68.93, and 71.70%, respectively, while, nitric oxide significantly decreased by 56.84, 75.78, and 63.15%, respectively. Similarly, superoxide dismutase significantly decreased by 86.45, 82.06, and 80.72%. In the same manner, glutathione level decreased by 70.63, 70.47, and 71.56%, respectively. Malondialdehyde also decreased by 51.56, 46.87, and 39.84%, while the total protein content decreased by 22.56, 16.99, and 30.71%, respectively (Table *S5* and Fig. 3b).

##### Cell organelles marker enzymes

The present study revealed insignificant changes in SDH, LDH, G-6-Pase, AP and 5′-Nu in normal rats protected with the plant extracts and ranitidine drug as compared with the control group (Table *S6* and Fig. 3c) in concerning cell organelles marker enzymes. Gastroulcerative rats showed significant increase in SDH, LDH, G-6-Pase, AP and 5′-Nu activities by 209.27, 390.29, 102.03, 172.04 and 116.56%, respectively as comped with the control group. Ulcerative rats protected with plant extracts and ranitidine drug recorded significant decrease in the selected enzyme markers with variable degrees as compared with the ulcerative group. SDH showed significant decrease by 78.86, 71.87and 71.60% after protection with leaves and bark of hybrid Abdel Razek, and ranitidine drug, respectively. LDH recorded inhibition activity by 76.64, 74.53, and 79.23%, while G-6-Pase showed inhibition by 40.50, 35.13, 37.56, 32.84 and 37.16%, respectively. In addition, AP enzyme decreased by 51.56, 59.22, and 52.85%, while 5′-Nu decreased by 47.12, 44.68, and 50.27%, respectively.

#### In vivo therapeutic effect of *Annona* Abdel Razek

##### Ulcer markers

**Fig. 4:**
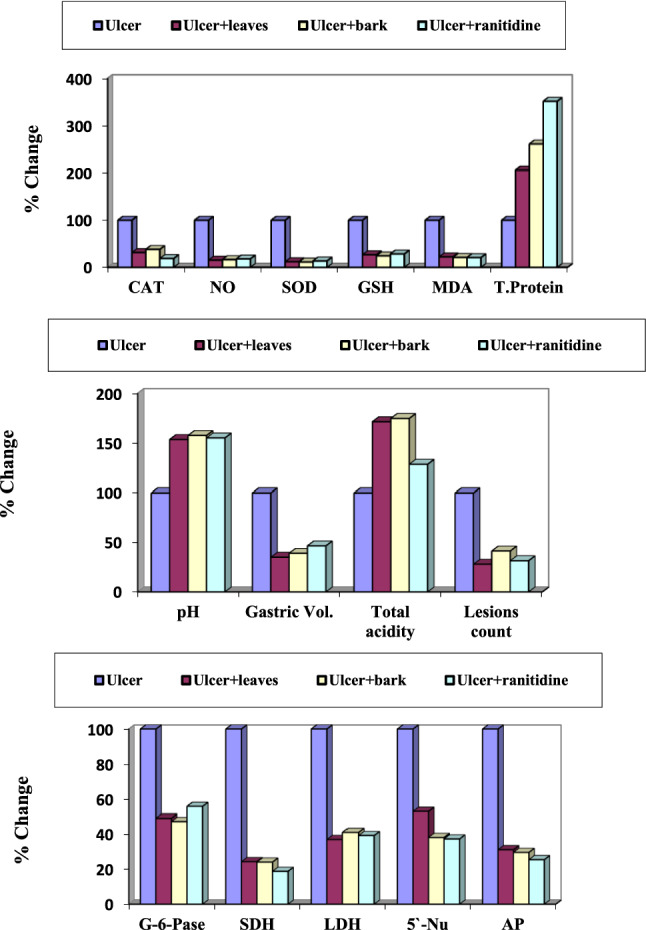
Percentage changes over ulcer group of the therapeutic effect of *A.* Abdel Razek extracts and ranitidine on **a** gastric ulcer markers, **b** antioxidant and protein levels and **c** cell organelles marker enzymes

In the therapeutic effect of the plant extract under investigation, Stomach pH in control rats treated with plant extracts did not change significantly, suggesting extract safety. Gastro-ulcerative rats showed significant decrease by 38.46% as compared with the control group. After treated with leaves and barks Abel Razek extracts as well as ranitidine drug, it showed significant increase in the pH level by 53.50, 57.50, and 55.00%, respectively when compared with the ulcer group (Table *S7* and Fig. 4a). Gastric volume in control rats treated with Abdel Razek extracts and the selected drug, recorded insignificant changes. Ulcerative stomach showed significant increase in its gastric volume content by 185.18% when compared with the control group. Treated rats with leaves and bark of hybrid Abdel Razek, and ranitidine drug recorded significant decrease in gastric volume by 64.90, 60.92, and 53.31%, respectively when compared with the ulcer group (Table *S7* and Fig. 4a). Normal rats treated with plant extracts recorded insignificant changes in gastric total acidity when compared with the control group (Table S7). The gastric ulcerative rats showed significant decrease in the total acidity values by 43.90% when compared with the control group. Treated rats with leaves and barks of Abdel Razek, and rantidine drug recorded significant increase in the total acidity level by 71.42, 74.69, and 28.57%, respectively comparing these values with those of the ulcer group (Table *S7* and Fig. 4a). The stomach of ulcerative group showed nearly twenty one ulcer lesions per stomach. Combined treatment with ethanol and two extracts or ranitidine reduced the number of lesions/organ by 71.92%, 58.55%, and 68.41%, respectively compared with the ulcerative group. (Table *S7* and Fig. 4a).

##### Antioxidant and total protein levels

Rats treated with Abdel Razek extracts or ranitidine drug showed not significant changes in catalase, NO, SOD, glutathione, malondialdehyde and total protein levels comparison with the control group. Significant increase in catalase, NO, SOD, glutathione, malondialdehyde and total protein levels in gastroulcerative rats by 130.23, 732.00, and 558.97 and 61.41%, were observed as compared with the control group, respectively. Similarly, decreased impacts of gastro-ulcerative rats with leaves and barks Abdel Razek and ranitidine drug were observed for nitric oxide 84.80%, 83.65%, and 82.21%; superoxide dismutase, 88.16%, 88.77%, and 86.75%; glutathione level, 73.36%, 75.81%, and 71.76%; malondialdehyde, 77.82, 79.18, and 79.37%, respectively. In contrast, total protein content significantly increased − 105.26%, 160.52%, and 251.26%, respectively (Table *S*8 and Fig. 4b).

##### Stomach marker enzymes

Insignificant changes were observed in SDH, LDH, G-6-Pase, AP and 5′-Nu enzymes in normal rats treated with leaves and barks of the plant extract under investigation as well as the reference drug in comparison with the control group. Gastroulcerative rats showed significant increase in SDH, LDH, G-6-Pase, AP and 5′-Nu activities by 287.16, 199.50, and 183.55%, respectively versus to the control group. Ulcerative rats treated with Abdel Razek leaves and barks extracts and ranitidine recorded significant decrease in the selected enzymes by variable degrees as compared with the ulcer group. SDH showed significant decrease by 75.61, 75.90, and 81.20%, respectively. Similarly, LDH, G-6-Pase, and AP enzymes decreased by 62.98%, 58.92, and 60.71%; 50.90%, 52.79%, and 44.27%; and 68.81%, 70.37%, and 74.49%, respectively while 5′-Nu decreased by 47.12, 61.80, and 62.63%, respectively (Tables *S*9 and Fig. 4c).

### Stomach histopathological features

#### Protective effect of *Annona* Abdel Razek

**Fig. 5:**
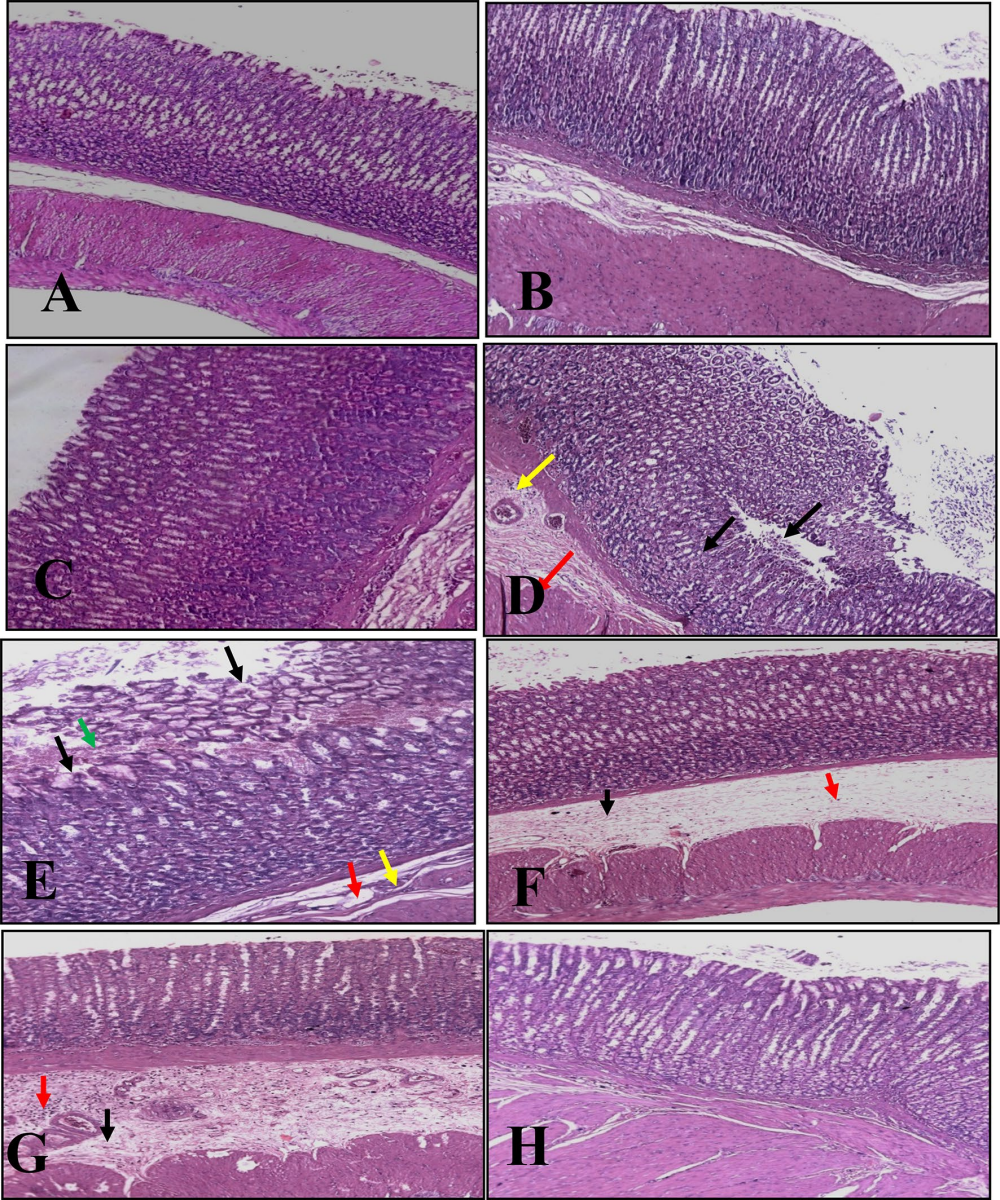
Photograph of rat stomach mucosal layers. **A** Normal rat stomach showing no damage to the mucosa and normal mucosal and submucosal layers (H&Ex100). **B **&** C** Normal rats treated with Abdel Razek's leaves and barks, respectively, showing minimal damage of the surface epithelium and no leucocyte infiltration of the submucosal layer (H&E stain 10 ×). **D** Stomach mucosa after one hour of ethanol ulceration, demonstrating erosion of the surface epithelium (black arrow), moderate edoema (red arrow), and moderate leucocyte infiltration of the submucosal layer (yellow arrow) (H&E stain 10 ×). **E** Stomach mucosa after one hour of ethanol ulceration, revealing erosion of the surface epithelium (black arrow), mild edema (red arrow), mild leucocyte infiltration of the submucosal layer (yellow arrow), and hemorrhage (green arrow) (H&E stain 20 ×). **F** Ulcerative gastric mucosa treated with Abdel Razek leaves demonstrating minimal rupture of the surface epithelium (healed), considerable edema (black arrow), and minor leucocyte infiltration of the submucosal layer (red arrow) (H&E stain 10 ×). **G** Ulcerative stomach mucosa treated with Abdel Razek's barks, demonstrating no damage of the surface epithelium (healed), considerable edema (black arrow), and minor leucocyte infiltration of the submucosal layer (red arrow) (H&E stain 10 ×). **H** Ulcerative gastric mucosa treated with ranitidine, revealing no disruption of the surface epithelium (healed) and modest leucocyte infiltration of the submucosal layer (H&E stain 10 ×)

No lesions were seen in gastric mucosa, including in normal mucosal and submucosal layers in control rats. Treatment with leaves and bark extracts in normal rats produced no damage to surface epithelia and did not induce leukocyte infiltration into submucosal layers (Fig. 5A, B and C). Surface epithelium was eroded with moderate edema and moderate leucocyte infiltration of the submucosal layer with bleeding in a section of stomach mucosa exposed to ethanol (Fig. 5D). Gastric mucosa was protected by administration of extracts where no disruption of the surface epithelium (healed) with significant edema and minor leucocyte infiltration of the submucosal layer was observed on histological examination (Fig. 5E, F and G).

#### Therapeutic effect of *Annona* Abdel Razek

**Fig. 6:**
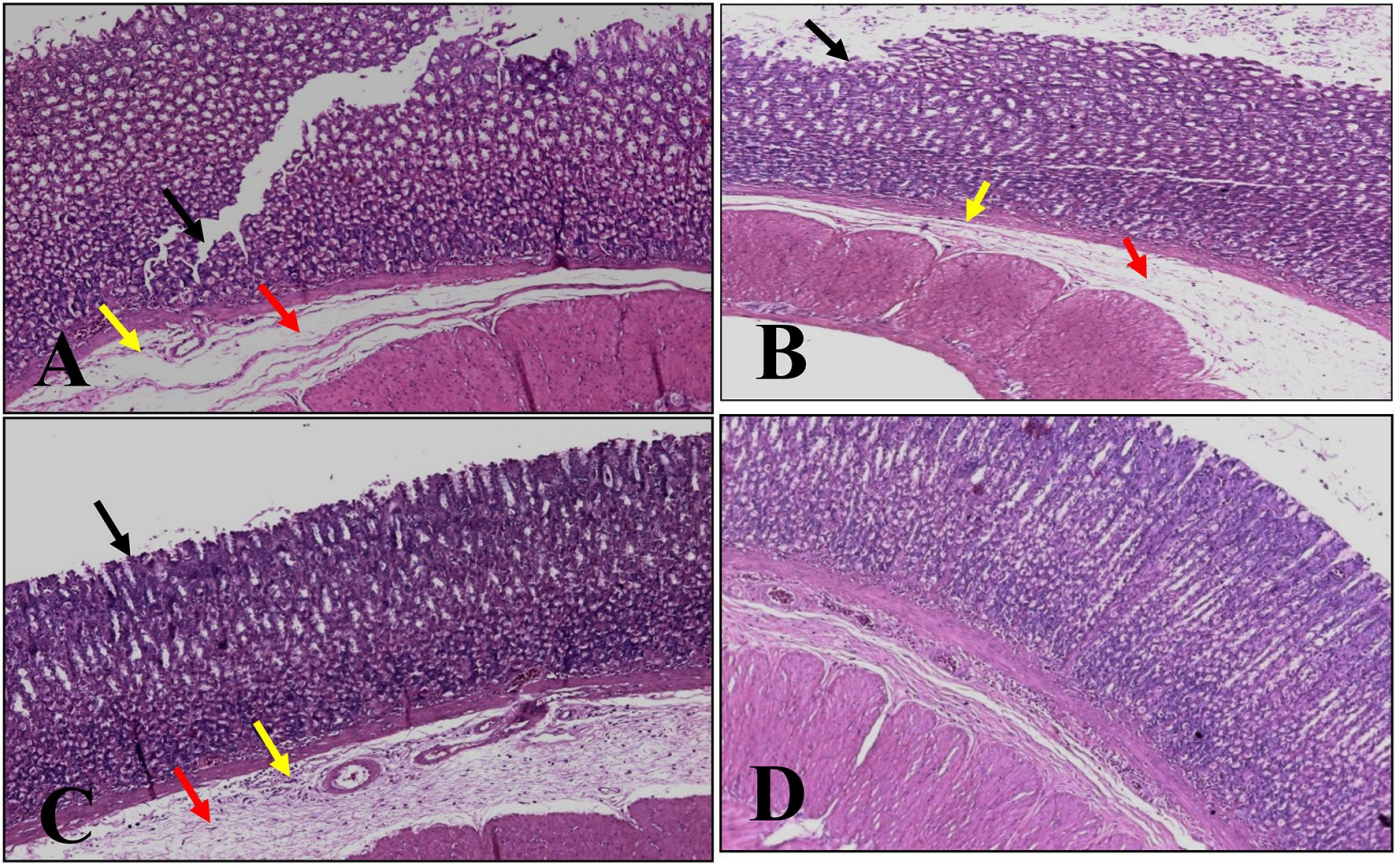
Photograph of rat stomach mucosal layers. **A** Gastric mucosa of ethanol induced ulcer for one week showing deep ulcer with surface epithelium (black arrow) with moderate edema (red arrow) and leucocytes infiltration of the submucosal layer (yellow arrow) (H&E stain 10 ×). **B** Ulcerative gastric epithelium pretreated with Abdel Razek leaves revealing surface epithelial degradation (black arrow), mild-moderate edema (red arrow), and minor leucocyte infiltration of the submucosal layer (yellow arrow) (H&E stain 10 ×). **C** Ulcerative gastric mucosa treated with Abdel Razek barks shows intact (healed) surface epithelium (black arrow), modest edema (red arrow), and mild leucocyte infiltration of the submucosal layer (yellow arrow) (H&E stain 10 ×). **D** Ulcerative gastric mucosa treated with ranitidine, revealing no rupture of the surface epithelium (healed), no edema, and no leukocyte infiltration of the submucosal layer (H&E stain 10 ×).

A histological section of stomach mucosa one week after ethanol administration (H&E stain 10x) shows a deep ulcer with surface epithelium (black arrow), considerable edema (red arrow), and leucocyte infiltration into the submucosal layer (yellow arrow) (Fig. 6A). Surface epithelium was eroded with mild to moderate edema and minor leucocyte infiltration into the submucosal layer in ulcerative gastric mucosa treated with Abdel Razek leaves (Fig. 6B). After administration of bark extract, surface epithelium of ulcerative gastric mucosa was intact with slight edema and minor leucocyte infiltration of the submucosal layer (Fig. 6C). The ulcerative stomach mucosa treated with ranitidine appeared normal on histological examination (Fig. 6D).

## Discussion

A combination of RAPD and ISSR data indicates 100% similarity between *A. cherimola* and *Annona* Abdel Razek and 80% between *A. squamosa* and *Annona* Abdel Razek. Sixty-three secondary metabolites were detected in leaves and bark of the new cultivar, *Annona* Abdel Razek, using UPLC high-resolution MS for simultaneous chromatographic separation and mass spectrometric detection. Phytochemical studies revealed the presence of alkaloids identified as liriodenine, lanuginosine, oxostephanosine, stepharine, coclaurine, corytuberine, fenfangjine G, oxoanolobine and phanostenine. These compounds are belonging to the oxoaporphine, aporphine, isoquinolines, and benzylisoquinoline groups. Alkaloids include a basic nitrogen atom and exhibit antiulcer properties. The antiulcer activity of 2-phenylquinoline is demonstrated in several pharmacological investigations that used EtOH to induce (Leite et al., 2020). Malonyl-CoA and p-coumaroyl-CoA are two fundamental metabolites that give flavonoids their fifteen-carbon skeletons (C6–C3–C6). The condensation of three molecules of malonyl-CoA with one molecule of p-coumaroyl-CoA produce a chalcone intermediate, which is key to the biosynthetic process. Chalcones are the building blocks for the wide variety of flavonoid derivatives found in plants (de Lira Mota et al., 2009). Several flavonol glycosides, including kaempferol, quercetin, and isorhamnetin, were found in the leaves and bark of *Annona* Abdel Razek. Phenolic acids (p-coumaric, ferulic, and caffeic) and their quinic acid derivatives were also identified as chemicals with antioxidant properties.

Ethanol destabilizes the mucus-bicarbonate-phospholipid layer, resulting in retro-diffusion of H + ions and damage to epithelial cells. This damage leads to altered levels of mucosal enzymes, such as SDH, LDH, G-6-Pase, AP and 5′-Nu. Mast cell activation causes the release of inflammatory mediators that encourage neutrophils to migrate to injured areas. Subsequently, generation of ROS increases cellular damage and causes tissue necrosis (Fahmi et al., 2019). Stomach ulcers are also affected by stomach acidity, and controlling acidity is a substantial challenge (Aboul Naser et al., 2020). Antiacids, H_2_ receptor blockers, such as ranitidine and its analogues, anticholinergics (pirenzepine), and proton pump blockers, such as omeprazole, were all employed (Wallace & Sharkey, 2011). However, most currently available medications have limited efficacy and cause adverse side effects, making stomach ulcer therapy a major concern.

Natural products have a significant role in controlling gastric ulcer disease (Newman & Cragg, 2020), reflecting their notable structural diversity and selective biological activity (Cragg & Newman, 2013). Flavonoids play an essential role in preventing gastric lesions caused by various ulcerogenic agents. They also aid in healing such ulcers and may be novel agents for suppressing peptic ulcers (de Lira Mota et al., 2009). Several reports on *Annona* species (Ma & Liu, 2014) support our current results that indicate that extracts of *A*nnona Abdel Razek leaves and bark may protect gastromucosal integrity via synergistic actions of flavonoids, condensed tannins, and saponins, in addition to alkaloids. Further, isocorydine, liriodenine, lanuginosine, oxostephanosine, stepharine, and coclaurine have all been identified as aporphine benzylisoquinolinic alkaloids. These alkaloids have been isolated from a variety of plant parts and species, including *Annona squamosa* twigs (Yadav et al., 2011), *Isopyrum thalictroides* (Ranunculaceae) roots and rhizomes (Istatkova & Philipov, 2004), and *Dactylicapnos scandens* (Papaveraceae) roots (Wang et al., 2018).

Qualitative and quantitative differences in phenolic, flavonoid and alkaloid constituents exist between *A. cherimolia* and *A. squamosa* and constituents identified in the present work. Current results are most similar to data reported by Yadav et al., 2011, who indicated that antisecretory mechanisms of isolated aporphine alkaloids from twigs *A. squamosa* such as N-methylcorydaldine, lanuginosine, ( +)-anomuricine, and N-methyl-6,7-dimethoxyisoquinolone, involve inhibition of gastric H^+^/ K^+^-ATPase activity.

Gastro-duodenal ulcers are associated with marked alterations in multiple biochemical parameters (Esmaeilnejad et al., 2012).A substantial increase in MDA, SOD, CAT, NO, and GSH was observed in the current study. Gastric mucosal MDA levels in patients with peptic ulcer and gastritis are likely to indicate free radical-mediated gastric mucosal damage (Ren et al., 2021). Hydroxyl radicals oxidize essential cellular structural and functional proteins, as well as membrane lipids. The loss of membrane fluidity, ion transport, integrity, and loss of various cellular functions, are all caused by lipid peroxidation (Aboul Naser et al., 2020). Glutathione levels are dependent on the activity of several enzymes (Rahman et al., 2020). For example, an increase in GSH concentration can be caused by the upregulation of γ -glutamyl-cysteine synthetase, an enzyme involved in GSH synthesis. Increased activity of GSH peroxidase and GSH transferase, however, reduces GSH concentration. Thus, a decrease in glutathione peroxidase and glutathione transferase is reported in indomethacin-induced stomach ulcers (Koc et al., 2008), ethanol-induced mucosal injury (Dejban et al., 2020), and stress ulcers (Liu et al., 2011).

The efficacy of antisecretory and antiulcerogenic actions of selected extracts is supported by an observed decrease in gastric volume, lesion count, and pH (Mohammed et al., 2020). Also, total protein levels can be used as an indicator of the severity of cellular dysfunction in a variety of diseases. Protein synthesis stimulation has been identified as a contributory self-healing mechanism that encourages regeneration (Bryndin & Bryndina, 2020).

The histological sections showed altered gastric mucosa after ethanol ulceration, with deep ulcers reaching the basement membrane of the *lamina propria*. Some polymorphous lymphocyte fibrin was found at the swollen ulcer base (Okabe & Pfeiffer, 1972). The ulcer is bordered by hyperplastic gastric glands. Few lymphocytes and polymorphonuclear leucocytes with a high degree of fibrosis are found in the *lamina propria*. Our histological observations of the ulcerative mucosa are consistent with these findings. We also noticed a well-developed epithelium at the ulcer margin in rats protected or treated with plant extracts. Interestingly, ulcer healing occurs either by a slow process involving cell migration to the luminal surface and deposition on the area stripped by an ulcerogenic agent or through a rapid process involving cell migration to the mucosal surface and deposition on areas reduced by the ulcerogenic agent (Joshi et al., 2011).

## Conclusion

The metabolomics profile of crude extracts of *Annona* Abdel Razek contains 63 compounds, including flavonoids, phenolic compounds, amino acids, and alkaloids. *Annona* Abdel Razek leaves and bark extracts exhibit anti-ulcerogenic properties. The bark extract shows better protective and healing activity for gastric ulcers than leaves extract. Further, the bark extract is more efficacious for therapy rather than prophylaxis.

## Supplementary Information

Below is the link to the electronic supplementary material.

Supplementary file1 (DOCX 877 kb)

## Data Availability

Data availability statements provide a statement about where data supporting the results reported in a published article can be found, including datasets analyzed or generated during the study.

## Abbreviations

UPLC: Ultra performance liquid chromatography
LCMSMS: Liquid chromatography–mass spectrometry
HPLC: High performance liquid chromatography
HESI: Heated Electrospray Ionization
ESI: Electrospray ionization
CID: Collision-induced dissociation
EI: Electron ionization
ABTS^+^: 2,2'-azino-bis(3-ethylbenzothiazoline-6-sulfonic acid)
DPPH: 2,2-diphenyl-1-picrylhydrazyl
