# Supplementary material for: Profiling of secondary metabolites and DNA typing of three different Annona cultivars grown in Egypt
Source: Metabolomics. 2022 Jul 4;18(7):49. doi: 10.1007/s11306-022-01911-w (PMC9252975; doi:10.1007/s11306-022-01911-w)
Supplement: Supplementary file 1 — Supplementary file1 (DOCX 877 kb) [file 11306_2022_1911_MOESM1_ESM.docx]

**Title:** Profiling of secondary metabolites and DNA typing of three different *Annona* cultivars grown in Egypt

Mona Arafa Mohammed^1*^, Manal A. Hamed^2^, Souad Eisawy El-Gengaihi^1^, Ahmed Mahmoud Aboul Enein^3^, Piotr Kachlicki^4^, Emad Mohamed Hassan^1^

^1^Medicinal and Aromatic Plants Research Dept., Pharmaceutical and Drug Industries Research Institute, National Research Centre, Dokki, 12311, Cairo, Egypt.

^2^ Department of Therapeutic Chemistry, Pharmaceutical and Drug Industries Research Institute, National Research Centre, Dokki, 12311, Cairo, Egypt.

^3^Biochemistry Department, Faculty of Agriculture, Cairo, University, Giza, Egypt.

^4^Institute of Plant Genetics of the Polish Academy of Sciences (Metabolomics group), Poznan, Poland.

* Corresponding authors:

Mona Arafa Mohammed^1*^ [monaarafamohammed@yahoo.com](mailto:monaarafamohammed@yahoo.com),

[on.ibrahim@nrc.sci.eg](mailto:on.ibrahim@nrc.sci.eg), tel. +201004800386, 0000-0002-9835-213X

Manal A. Hamed^2*^ [manal_hamed@yahoo.com](mailto:manal_hamed@yahoo.com), tel. +2 01001298522

1. **MATERIALS**
   1. **Polymerase chain reaction (PCR) condition stock solutions [5x Tris-borate (TBE), pH 8.0]**

| Tris-base | 5.40 g |
| --- | --- |
| Boric acid | 2.75 g |
| 500 mM EDTA, 8.0 | 0.29 g |
| H_2_O (d.w) up to | 1. l |

- 1. **Ethidium bromide**

The stock solution was prepared by dissolving 1 g of ethidium bromide in 100 ml distilled water and mixed well with magnetic stirrer then transferred to a dark bottle and stored at room temperature.

- 1. Sample loading dye (5x)

| Na-EDTA, pH 8.0 (500 mM) | 2.00 ml |
| --- | --- |
| Glycerol (100%) | 5.00 ml |
| Bromophenol blue (2%) | 0.75 ml |
| H_2_O (d.w.) | - 1. ml |

- 1. **PCR was performed in 30-µl volume tubes according to (Williams, Kubelik, Livak, Rafalski, Tingey 1990) that contained the following:**

| dNTPs (2.5 mM) | 3.00 µl |
| --- | --- |
| MgCl_2_ (25 mM) | 3.00 µl |
| Buffer (10 x) | 3.00 µl |
| Primer (10 pmol) | 2.00 µl |
| Taq DNA polymerse (5U/µl) | 0.20 µl |
| Template DNA (25 ng) | 2.00 µl |
| H_2_O (d.w.) | - 1. l |

**1.5. Gel preparation procedure**

1. Agarose (1.50 gm) was mixed with (100ml) l x TBE buffer and boiled in microwave.

2. Ethidium bromide (5µl) was added to the melted gel after the temperature became 55°C.

3. The melted gel were poured in the tray of mini-gel apparatus and comb was inserted immediately, then comb was removed when the gel become hardened.

4. The gel was covered by the electrophoretic buffer (1x TBE).

5. DNA amplified product (15 µl) was loaded in each well.

6. DNA ladder (100bpp) mix was used as standard DNA with molecular weights of 1500,1000, 900, 800, 700, 600, 500, 400, 300, 200 and 100 bp. The run was performed for about 30 min at 80 V in mini submarine gel BioRad.

1. **METHODS**

**2.1.** [**DNA fingerprinting**](http://www.britannica.com/EBchecked/topic/167155/DNA-fingerprinting)

**2.1.1. DNA isolation procedure**

Fresh tissue parts were collected separately from three different *Annona* species. The bulked DNA extraction was performed using Spin Collumn Kit (Biobasic.Com.). Isolation protocol of DNA (Dellaporta, Wood, Hicks 1983) was as follows:

Buffer AP1 (400 µl) and 4 µl of RNase a stock solution (100 mg/ml) and 5 µl proteinase K were added to a maximum of 400 ml of tissues and vortexed vigorously. Mixture was incubated for 10 min at 65^o^C and mixed 2-3 times during incubation by inverting tube. Then, 130 µl of buffer AP2 was added to the lysate, mixed and incubated for 5 min on ice. Lysate was applied to the QIA shredder spin column sitting in a 2 ml collection tube and centrifuged for 2 min at maximum speed (10.000) rpm. Supernatant from step 4 was transferred to a new tube without disturbing the cell-debris pellet. Typically, 450 µl of lysate was recovered. 0.5 volume of buffer AP3 and 1 volume of ethanol (96-100%) were added to the cleared lysate and mixed by pipetting. 650 µl of the mixture from step 6 was applied through DNeasy Mini spin column setting in a 2 ml collection tube, then, centrifuged for 1 min at 8000 rpm and flow-through was then discarded. DNeasy column was then placed in a new 2 ml collection tube. Then, 500 µl buffer AW was added onto the DNeasy column and centrifuged for 1 min at 8000 rpm. 500 µl buffer AW was added to DNeasy column and centrifuged for 2 min at maximum speed (10.000 rpm) to dry the column membrane. DNeasy column was then transferred to a 1.5 ml microfuge tube and 100 µl of preheated (65^o^C) buffer AE was pipetted directly onto the DNeasy column membrane. Then, incubated for 5 min at room temperature and centrifuged for 1 min at 8000 rpm to elute. Elution was repeated once as described. A new microfuge can be used for first elute. Alternatively, the microfuge tube can be reused for the second elution step to combine the elutes.

**2.2.2. RAPD -PCR Polymerase Chain Reaction Analysis and ISSR -PCR Analysis**

In order to obtain clear reproducible amplification products, different preliminary experiments were carried out in which a number of factors were optimized. These factors included PCR temperature cycle profile and concentration of each of the template DNA, primer, MgCl_2_ and Taq polymerase. A total of twenty-one random DNA oligonucleotide primers were independently used according to Williams *et al*. (1990) in the PCR reaction. Only five primers succeeded to generate reproducible polymorphic DNA products. Table (1) lists the base sequences of these DNA primers that produced informative polymorphic bands.

The PCR amplification was performed in a 25 µl reaction volume containing the following: 2.5 µl of dNTPs (2.5 mM), 1.5µl of Mg Cl_2_ (25 mM), 2.5 µl of 10x buffer, 2.0 µl of primer (2.5 µM), 2.0 µl of template DNA (50 ng/µl), 0.3 µl of Taq polymerase (5 U/µl) and 14.7 µl of sterile ddH_2_O. The reaction mixtures were overlaid with a drop of light mineral oil per sample. Amplification was carried out in Techne TC-512 PCR System. The reaction was subjected to one cycle at 95ºC for 5 minutes, followed by 35 cycles at 94 ºC for 30 seconds, 37 ºC for 30 seconds, and 72 ºC for 30 seconds, then a final cycle of 72 ºC for 12 minutes. PCR products were run at 100 V for one hour on 1.4 % agarose gels to detect polymorphism between virus strains under study. After electrophoresis, the RAPD patterns were visualized with UV transilluminator.

PCR amplification was performed using seven random 10 mer arbitrary primers synthesized by (Operon biotechnologies, Inc.Germany) with the following sequences. After electrophoresis, the ISSR patterns were visualized with UV transilluminator. ISSR markers were scored from the gels as DNA fragments present or absent in all lanes (Tables *S*1-*S*3).

**Table *S*1. Distribution of RAPD markers in the studied samples**

| **Primers** | | | | | |
| --- | --- | --- | --- | --- | --- |
| **OP-A01** | | | | | |
| **Band No.** | **M.W(bp)** | **Cultivars** | | | **Polymorphism** |
|  |  | **1** | **2** | **3** |  |
| 1 | 1200 | 1 | 0 | 1 | Polymorphic |
| 2 | 780 | 1 | 1 | 1 | Monomorphic |
| 3 | 655 | 0 | 1 | 1 | Polymorphic |
| 4 | 560 | 0 | 1 | 1 | Polymorphic |
| 5 | 460 | 1 | 1 | 1 | Monomorphic |
| 6 | 410 | 1 | 1 | 1 | Monomorphic |
| 7 | 325 | 1 | 1 | 1 | Monomorphic |
| 8 | 270 | 0 | 1 | 1 | Polymorphic |
| **Total** |  | **5** | **7** | **8** |  |
| **OP-B02** | | | | | |
| **Band No.** | **M.W.(bp)** | **Cultivars** | | | **Polymorphism** |
|  |  | **1** | **2** | **3** |  |
| 1 | 1390 | 1 | 0 | 0 | Polymorphic |
| 2 | 1020 | 1 | 0 | 0 | Polymorphic |
| 3 | 920 | 1 | 0 | 0 | Polymorphic |
| 4 | 780 | 1 | 0 | 0 | Polymorphic |
| 5 | 625 | 1 | 0 | 0 | Polymorphic |
| 6 | 470 | 0 | 1 | 1 | Polymorphic |
| 7 | 445 | 1 | 0 | 1 | Polymorphic |
| 8 | 390 | 1 | 1 | 1 | Monomorphic |
| 9 | 335 | 1 | 0 | 1 | Polymorphic |
| 10 | 290 | 1 | 0 | 0 | Polymorphic |
| 11 | 260 | 1 | 1 | 1 | Monomorphic |
| **Total** |  | **10** | **3** | **5** |  |
| **OP-B07** | | | | |  |
| **Band No.** | **M.W.(bp)** | **Cultivars** | | | **Polymorphism** |
|  |  | **1** | **2** | **3** |  |
| 1 | 1350 | 1 | 1 | 1 | Monomorphic |
| 2 | 1150 | 1 | 1 | 1 | Monomorphic |
| 3 | 900 | 1 | 1 | 1 | Monomorphic |
| 4 | 780 | 1 | 1 | 1 | Monomorphic |
| 5 | 635 | 1 | 1 | 1 | Monomorphic |
| 6 | 530 | 1 | 1 | 1 | Monomorphic |
| 7 | 450 | 1 | 0 | 0 | Polymorphic |
| 8 | 385 | 1 | 1 | 1 | Monomorphic |
| 9 | 305 | 1 | 1 | 0 | Polymorphic |
| 10 | 250 | 1 | 1 | 1 | Monomorphic |
| 11 | 155 | 1 | 1 | 1 | Monomorphic |
| **Total** |  | **11** | **10** | **9** |  |
| **OP-B11** | | | | |  |
| **Band No.** | **M.W.(bp)** | **Cultivars** | | | **Polymorphism** |
|  |  | **1** | **2** | **3** |  |
| 1 | 1455 | 0 | 0 | 1 | Polymorphic |
| 2 | 1095 | 1 | 0 | 0 | Polymorphic |
| 3 | 825 | 1 | 1 | 1 | Monomorphic |
| 4 | 770 | 1 | 0 | 0 | Polymorphic |
| 5 | 660 | 1 | 1 | 1 | Monomorphic |
| 6 | 495 | 1 | 1 | 1 | Monomorphic |
| 7 | 410 | 1 | 0 | 1 | Polymorphic |
| 8 | 330 | 1 | 0 | 0 | Polymorphic |
| 9 | 315 | 0 | 0 | 1 | Polymorphic |
| 10 | 290 | 1 | 0 | 0 | Polymorphic |
| 11 | 225 | 1 | 0 | 0 | Polymorphic |
| **Total** |  | **9** | **3** | **6** |  |
| **OP-C04** | | | | |  |
| **Band No.** | **M.W.(bp)** | **Cultivars** | | | **Polymorphism** |
|  |  | **1** | **2** | **3** |  |
| 1 | 680 | 1 | 0 | 1 | Polymorphic |
| 2 | 575 | 1 | 0 | 0 | Polymorphic |
| 3 | 485 | 1 | 0 | 1 | Polymorphic |
| 4 | 350 | 1 | 0 | 0 | Polymorphic |
| 5 | 270 | 1 | 1 | 1 | Monomorphic |
| 6 | 240 | 1 | 1 | 1 | Monomorphic |
| 7 | 180 | 1 | 1 | 1 | Monomorphic |
| **Total** |  | **7** | **3** | **5** |  |

1= *Annona squamosa*,

2= *Annona cherimola* and

3= *Annona Abdel Razek* (the hybrid)

**Table S2. Distribution of ISSR markers in the studied samples.**

| **Primers** | | | | | | | | | |
| --- | --- | --- | --- | --- | --- | --- | --- | --- | --- |
| **14 A** | | | | | | | | | |
| **Band No.** | | **M.W (bp)** | **Cultivars** | | | | | | **Polymorphism** |
|  | |  | **1** | | **2** | | **3** | |  |
| 1 | | 820 | 0 | | 0 | | 1 | | Polymorphic |
| 2 | | 580 | 1 | | 1 | | 1 | | Monomorphic |
| 3 | | 530 | 1 | | 0 | | 1 | | Polymorphic |
| 4 | | 440 | 1 | | 1 | | 1 | | Monomorphic |
| 5 | | 390 | 1 | | 1 | | 1 | | Monomorphic |
| 6 | | 295 | 1 | | 1 | | 1 | | Monomorphic |
| **Total** | |  | **5** | | **4** | | **6** | |  |
| **44B** | | | | | | | | | |
| **Band No.** | | **M.W (bp)** | **Cultivars** | | | | | | **Polymorphism** |
|  | |  | **1** | | **2** | | **3** | |  |
| 1 | | 640 | 1 | | 1 | | 1 | | Monomorphic |
| 2 | | 560 | 1 | | 1 | | 1 | | Monomorphic |
| 3 | | 490 | 1 | | 1 | | 1 | | Monomorphic |
| 4 | | 440 | 0 | | 1 | | 1 | | Polymorphic |
| 5 | | 400 | 1 | | 1 | | 1 | | Monomorphic |
| 6 | | 370 | 1 | | 1 | | 1 | | Monomorphic |
| 7 | | 320 | 1 | | 1 | | 1 | | Monomorphic |
| 8 | | 280 | 1 | | 0 | | 0 | | Polymorphic |
| **Total** | |  | **7** | | **7** | | **7** | |  |
| **HB-08** | | | | | | | | |  |
| **Band No.** | | **M.W (bp)** | **Cultivars** | | | | | | **Polymorphism** |
|  | |  | **1** | | **2** | | **3** | |  |
| 1 | | 825 | 0 | | 1 | | 1 | | Polymorphic |
| 2 | | 480 | 0 | | 1 | | 0 | | Polymorphic |
| 3 | | 420 | 0 | | 1 | | 0 | | Polymorphic |
| 4 | | 325 | 0 | | 1 | | 0 | | Polymorphic |
| 5 | | 300 | 1 | | 0 | | 0 | | Polymorphic |
| 6 | | 270 | 0 | | 0 | | 1 | | Polymorphic |
| 7 | | 250 | 1 | | 1 | | 0 | | Polymorphic |
| 8 | | 245 | 0 | | 0 | | 1 | | Polymorphic |
| 9 | | 145 | 1 | | 1 | | 0 | | Polymorphic |
| **Total** | |  | **3** | | **6** | | **3** | |  |
| **HB-11** | | | | | | | | |  |
| **Band No.** | | **M.W (bp)** | **Cultivars** | | | | | | **Polymorphism** |
|  | |  | **1** | | **2** | | **3** | |  |
| 1 | | 1040 | 1 | | 0 | | 0 | | Polymorphic |
| 2 | | 1000 | 0 | | 0 | | 1 | | Polymorphic |
| 3 | | 920 | 1 | | 1 | | 0 | | Polymorphic |
| 4 | | 865 | 0 | | 0 | | 1 | | Polymorphic |
| 5 | | 810 | 1 | | 1 | | 1 | | Monomorphic |
| 6 | | 740 | 0 | | 1 | | 0 | | Polymorphic |
| 7 | | 700 | 1 | | 0 | | 1 | | Polymorphic |
| 8 | | 640 | 1 | | 1 | | 1 | | Monomorphic |
| 9 | | 440 | 1 | | 1 | | 1 | | Monomorphic |
| 10 | | 385 | 1 | | 1 | | 1 | | Monomorphic |
| 11 | | 330 | 0 | | 1 | | 0 | | Polymorphic |
| 12 | | 295 | 1 | | 1 | | 0 | | Polymorphic |
| **Total** | |  | **8** | | **8** | | **7** | |  |
| **HB-15** | | | | | | | | |  |
| **Band No.** | **M.W.(bp)** | | **Cultivars** | | | | | | **Polymorphism** |
|  |  | | **1** | **2** | | **3** | |  | |
| 1 | 1070 | | 1 | 1 | | 1 | | Monomorphic | |
| 2 | 980 | | 1 | 1 | | 1 | | Monomorphic | |
| 3 | 720 | | 1 | 1 | | 1 | | Monomorphic | |
| 4 | 660 | | 1 | 1 | | 1 | | Monomorphic | |
| 5 | 570 | | 1 | 1 | | 1 | | Monomorphic | |
| 6 | 475 | | 1 | 0 | | 0 | | Polymorphic | |
| 7 | 435 | | 0 | 1 | | 1 | | Polymorphic | |
| 8 | 340 | | 0 | 1 | | 1 | | Polymorphic | |
| **Total** |  | | **6** | **7** | | **7** | |  | |

1= *Annona squamosa*,

2= *Annona cherimola* and

3= *Annona Abdel Razek* (the hybrid)

**Table *S*3:**  Number of total bands, monomorphic bands and polymorphic bands and percentage of polymorphism revealed by the five 10-mer primers in the studied samples by RAPD.

| **Primers** | **No. of bands** | **Monomorphic bands** | **Polymorphic bands** | **Polymorphism %** |
| --- | --- | --- | --- | --- |
|  |  | **RAPD** |  |  |
| **OP-A01** | 8 | 4 | 4 | 50% |
| **OP-B02** | 11 | 2 | 9 | 81.82% |
| **OP-B07** | 11 | 9 | 2 | 18.18% |
| **OP-B11** | 11 | 3 | 8 | 72.73% |
| **OP-C04** | 7 | 3 | 4 | 57.14% |
| **Total** | **48** | **21** | **27** | **55.97%** |
|  |  | **ISSR** |  |  |
| **14A** | **6** | **4** | **2** | **33.33%** |
| **44B** | **8** | **6** | **2** | **25%** |
| **HB-08** | **9** | **0** | **9** | **100%** |
| **HB-11** | **12** | **4** | **8** | **66.67%** |
| **HB-15** | **8** | **5** | **3** | **37.5%** |
| **Total** | **43** | **19** | **24** | **Mean 52.50%** |

**Table *S*4:** Protective effect of Abdel Razek leaves and bark extracts on gastric ulcer index in different studied groups

| **Parameters** | **Control** | **Control+ Leaves extract** | **Control +Barks extract** | **Ulcer** | **Rats protected with leaves extract** | **Rats protected with barks extract** | **Rats protected with Ranitidine** |
| --- | --- | --- | --- | --- | --- | --- | --- |
| **pH** | 5.50 ± 0.46^b -^ | 5.37 ± 0.51^b^  (-2.36) | 5.37 ± 0.51^b^  (-2.36) | 3.25 ± 0.53^a^ (-40.90) | 5.25 ± 0.46^b^  (-4.54) | 5.25 ± 0.71^b^ (-4.54) | 5.62 ± 0.74^b^ (+2.18) |
| **Gastric volume** | 132.37 ± 18.18^e -^ | 147.37 ± 16.06^e^ (+11.33) | 146.00 ± 17.36^e^ (+10.99) | 3125.00 ± 18.22^a^ (+2260.80) | 1112.50 ± 17.68^d^  (+740.44) | 1331.25 ± 17.15^c^ (+905.70) | 2050.00 ± 29.60^b^  (+1448.68) |
| **Total acidity** | 2.51 ± 0.28 ^abcd -^ | 2.59 ± 0.30^abcd^ (-12.54) | 0.67 ± 0.22^e^  (-9.75) | 2.51 ± 0.28 ^abcd^ (-76.65) | 1.97 ± 0.62^cd^  (+194.00) | 2.21 ± 0.61^bcd^ (+229.00) | 1.82 ± 0.66^d^ (+171.64) |
| **Lesions count** | -  - | -  - | -  - | 11.25 ± 1.98^a^  - | 3.00 ± 0.534^bc^  [-73.33] | 1.75 ± 1.64^c^ [- 84.44] | 3.25 ± 0.88^b^  [-70.84] |

- Data are mean ± SD of eight rats in each group.
- Values are expressed as (µL) in Gastric Volume, (meq/L) in Total Acidity.
- Statistical analysis is carried out by one way analysis of variance (ANOVA), Co-stat Computer Program accompanied by lease significance difference (LSD) test between groups at p<0.05.
- Shared letters are non-significant values between groups.
- Unshared letters are significant values between groups at p<0.0001.
- Values between brackets are percentage changes over control group.
- Values between parentheses are percentage changes over ulcer group.

**Table *S*5:** Protective effect of Abdel Razek leaves and bark extracts on antioxidant and protein levels in different studied groups.

| **Parameters** | **Control** | **Control+ Leaves extract** | **Control +Barks extract** | **Ulcer** | **Rats protected with leaves extract** | **Rats protected with barks extract** | **Rats protected with Ranitidine** |
| --- | --- | --- | --- | --- | --- | --- | --- |
| **CAT** | 3.59 ± 1.39^cd^ - | 2.69 ± 0.98^de^ (-25.06) | 2.77 ± 1.79^de^ (-22.84) | 8.66 ± 1.10^a^ (+141.22) | 4.32 ± 1.58^bc^ (+20.33) | 2.69 ± 0.90^de^ (-25.06) | 2.45 ± 0.55^de^ (-31.75) |
| **NO** | 0.25 ± 0.03^cd^ - | 0.30 ± 0.06^bcd^ (+20.00) | 0.26 ± 0.06^cd^ (+4.00) | 0.95 ± 0.09^a^ (+280.00) | 0.41 ± 0.09^b^  (+6400) | 0.23 ± 0.061^d^ (-8.00) | 0.35 ± 0.10^bcd^  (+40.00) |
| **SOD** | 16.22 ± 4.94^b^  - | 21.60 ± 3.10^b^ (+32.92) | 25.75 ± 4.84^b^ (+58.46) | 147.21 ± 21.71^a^ (+805.90) | 19.94 ± 8.11^b^  (+22.93) | 26.40 ± 6.32^b^  (+62.46) | 28.37 ± 6.84^b^ (+74.58) |
| **GSH** | 25.50 ± 2.41^b^ - | 25.71 ± 1.44^b^ (+0.82) | 23.74 ± 1.90^b^  (-6.90) | 94.10 ± 12.00^a^ (+269.01) | 27.63 ± 1.52^b^ (+8.35) | 27.78 ± 1.19^b^ (+8.94) | 26.76 ± 1.16^b^ (+4.90) |
| **MDA** | 0.78 ± 0.02^bcd^  - | 0.74 ± 0.04^bcd^ (-5.12) | 0.71 ± 0.08^bcd^  (- 8.84) | 1.28 ± 0.15^a^  (+64.10) | 0.62 ± 0.16^d^ (-20.51) | 0.68 ± 0.08^bcd^ (-12.82) | 0.77 ± 0.21^bcd^ (-1.28) |
| **T. protein** | 24.62 ± 7.4 ^de^ - - | 25.37 ± 10.6^de^ (+3.04) | 25.5 ± 0.53^de^ (+3.57) | 38.25 ± 4.49^a^  (+55.36) | 29.62 ± 3.37^bc^ (+20.30) | 31.75 ± 2.05^b^ (+28.96) | 26.5. ± 2.20^d^ (+7.63) |

- Data are mean ± SD of eight rats in each group.
- Values are expressed as µmol/mg protein for CAT, Mmol/mg protein for NO, µmol/mg protein for SOD, µg/mg protein for GSH, µmol/mg protein for MDA and **as** mg/g tissue for T. Protein.
- Statistical analysis is carried out by one way analysis of variance (ANOVA), Co-stat Computer Program accompanied by lease significance difference (LSD) test between groups at p<0.05.
- Shared letters are non-significant values between groups.
- Unshared letters are significant values between groups at p<0.0001.
- Values between brackets are percentage changes over control group.

**Table *S*6:** Protective effect Abdel Razek leaves and bark extracts on cel organelles marker enzymes in different studied groups

| **Parameters** | **Control** | **Control+ Leaves extract** | **Control +Barks extract** | **Ulcer** | **Rats protected with leaves extract** | **Rats protected with barks extract** | **Rats protected with Ranitidine** |
| --- | --- | --- | --- | --- | --- | --- | --- |
| **G6P** | 242.03 ± 23.23^fg^  - | 229.76 ± 9.29^g^ (- 5.06) | 262.94± 19.86^efg^ (+8.63) | 488.93± 45.37^a^ (+102.03 | 290.91 ± 8.45^bcde^ (+20.19) | 317.13 ± 22.58^bc^ (+31.02) | 307.24 ± 15.74^bcd^  (+26.94) |
| **SDH** | 74.94 ± 12.82^c^  - | 75.64 ± 13.35^c^  (+0.93) | 76.50 ± 9.93^c^  (+2.08) | 231.77 ± 27.84^a^ (+209.27 | 48.99 ± 10.05^d^  (-34.62) | 65.19 ± 10.45^c^ (-13.01) | 65.81 ± 8.69^c^ (-12.18) |
| **LDH** | 54.61 ± 4.21^b^  - | 57.51 ± 3.52^b^  (+4.65) | 59.51 ± 6.41^b^ (+8.97) | 267.75 ± 24.66^a^ (+390.29 | 62.53 ± 6.45^b^ (+14.50) | 68.17 ± 17.14^b^ (+24.83) | 55.59 ± 10.92^b^ (+1.79) |
| **5^`^Nu** | 52.12 ± 5.30^c^  - | 51.65 ± 5.24^c^  (-0.901) | 47.39 ± 7.55^c^ (-9.07) | 112.35 ± 16.61^a^ (+116.56 | 59.41 ± 8.03^c^  (+13.98) | 62.15 ± 9.02^bc^ (+19.24) | 55.87 ± 6.82^c^ (+7.19) |
| **AP** | 73.32 ± 5.96^c^  - | 73.92 ± 7.60^c^  (+0.81) | 72.74 ± 8.24^c^ (- 0.79) | 199.46 ± 23.90^a^ (+172.04 | 86.05 ± 12.84^bc^ (+17.36) | 81.32 ± 6.87^bc^ (+10.91) | 94.03 ± 15.26^b^ (+28.24) |

- Data are mean ± SD of eight rats in each group.
- Values are expressed as µmol/mg protein in all organelles marker enzymes.
- Statistical analysis is carried out by one way analysis of variance (ANOVA), Co-stat Computer Program accompanied by lease significance difference (LSD) test between groups at p<0.05.
- Shared letters are non-significant values between groups.
- Unshared letters are significant values between groups at p<0.0001.
- Values between brackets are percentage changes over control group.

**Table *S*7:** Therapeutic effect of Abdel Razek leaves and bark extracts on gastric ulcer markers in different studied groups

| **Parameters** | **Control** | **Control+ Leaves extract** | **Control +Barks extract** | **Ulcer** | **Rats treated with leaves extract** | **Rats treated with barks extract** | **Rats treated with Ranitidine** |
| --- | --- | --- | --- | --- | --- | --- | --- |
| **pH** | 3.25 ± 0.46^ab -^ | 3.37 ± 0.51a^b^  (+3.69) | 3.37 ± 0.51a^b^ (+3.69) | 2.00 ± 0.53^c^ (-38.46) | 3.07 ± 0.51a^b^ (-5.53) | 3.15 ± 0.75^a^  (-3.07) | 3.10 ± 0.53^bc^ (-4.61) |
| **Gastric volume** | 132.37 ± 18.18^e^  ^-^ | 147.37 ± 16.06^e^ (+11.33) | 146.00 ± 17.36^e^ (+10.29) | 377.50 ± 44.32^a^ (+185.18) | 132.5 ± 15.5^c^ (+0.09) | 147.5 ± 10.35^c^ (+11.43) | 176.25 ± 10.60^b^ (+33.14) |
| **Total acidity** | 2.87 ± 0.50^a -^ | 2.51 ± 0.28 ^a^ (-12.54) | 2.59 ± 0.30^a^ (-9.75) | 1.61 ± 0.24^c^ (-43.90) | 2.76 ± 0.30^a^ (-3.83) | 2.82 ± 0.68^a^ (-1.74) | 2.07 ± 0.12^b^ (-27.87) |
| **Lesions count** | - | - | - | 21.37 ± 3.24^a -^ | 6.00 ± 2.00^b^ [-71.92] | 3.00 ± 1.4^c^  [- 58.55] | 6.75 ± 2.81^b^  [- 68.41] |

- Data are mean ± SD of eight rats in each group.
- Values are expressed as (µL) in Gastric Volume, (meq/L) in Total Acidity.
- Statistical analysis is carried out by one way analysis of variance (ANOVA), Co-stat Computer Program accompanied by lease significance difference (LSD) test between groups at p<0.05.
- Shared letters are non-significant values between groups.
- Unshared letters are significant values between groups at p<0.0001.
- Values between brackets are percentage changes over control group.
- Values between parentheses are percentage changes over ulcer group

**Table S8:** Therapeutic effect of Abdel Razek leaves and bark extracts on antioxidant and protein levels in different studied groups

| **Parameters** | **Control** | **Control +Leaves extract** | **Control +Barks extract** | **Ulcer** | **Rats treated with leaves extract** | **Rats treated with barks extract** | **Rats treated with Ranitidine** |
| --- | --- | --- | --- | --- | --- | --- | --- |
| **CAT** | 3.59 ± 1.39^bc^  - | 2.69 ± 0.98^bcd^  (-25.06) | 2.77 ± 1.79^bcd^ (-22.84 | 10.87 ± 2.90^a^ (+130.23) | 3.4 ± 0.85^bcd^ (-5.29) | 4.14 ± 1.4^d^ (+15.32) | 2.02 ± 1.45^cd^ (-43.73) |
| **NO** | 0.25 ± 0.03^b^ - | 0.30 ± 0.06^b^ (+20.00) | 0.26 ± 0.06^b^ (+4.00) | 2.08 ± 0.57^a^ (+732.00) | 0.316 ± 0.04^b^ (+62.40) | 0.34 ± 0.07^b^ (+36.00) | 0.37 ± 0.09^b^  (+48.00) |
| **SOD** | 16.22 ±4.94^b^ - | 21.60 ± 3.10^b^ (+32.92) | 25.75 ± 4.84^b^ (+58.46) | 218.35 ± 64.20^a^ (+1243.69) | 25.85 ± 7.63^b^ (+59.07) | 24.39 ± 5.48^b^  (+50.09) | 28.92± 7.22^b^  (+77.96) |
| **GSH** | 25.50 ±2.41^cd^  - | 25.71 ± 1.44^cd^ (+0.82) | 23.74 ± 1.90^d^ (-6.90) | 107.42 ± 5.39^a^  (+321.25) | 28.61 ± 3.44^b^ (+12.19) | 25.98 ± 1.78^cd^ (+1.88) | 30.33 ± 4.60^b^ (+18.94) |
| **MDA** | 0.78 ± 0.02^cd^ - - | 0.74 ± 0.04^cd^  (-5.12) | 0.71 ± 0.08^cd^ (- 8.84) | 5.14 ± 0.14^a^ (+558.97) | 1.14 ± 0.37^b^ (+46.15) | 1.07 ± 0.83^bc^ (+37.17) | 1.06 ± 0.57^bc^  (+35.89) |
| **T. protein** | 24.62 ± 7.4^de^ - | 25.37 ± 10.6^d^ (+3.04) | 25.5 ± 0.53^d^  (+3.57) | 9.5 ± 3.02^g^ (-61.41) | 19.5 ± 7.17^f^  (+20.79) | 24.75 ± 3.33^de^  (+0.52) | 33.75 ± 7.15^a^ (+37.08) |

- Data are mean ± SD of eight rats in each group.
- Values are expressed as µmol/mg protein for CAT, Mmol/mg protein for NO, µmol/mg protein for SOD, µg/mg protein for GSH, µmol/mg protein for MDA and **as** mg/g tissue for T. Protein.
- Statistical analysis is carried out by one way analysis of variance (ANOVA), Co-stat Computer Program accompanied by lease significance difference (LSD) test between groups at p<0.05.
- Shared letters are non-significant values between groups.
- Unshared letters are significant values between groups at p<0.0001.
- Values between brackets are percentage changes over control group.

**Table *S*9:** Therapeutic effect of Abdel Razek leaves and bark extracts on cll organelles marker enzymes in different studied groups.

| **Parameters** | **Control** | **Control+ Leaves extract** | **Control +Barks extract** | **Ulcer** | **Rats treated with leaves extract** | **Rats treated with barks extract** | **Rats treated with Ranitidine** |
| --- | --- | --- | --- | --- | --- | --- | --- |
| **G6P** | 242.03± 23.23^c^ - | 229.76 ± 9.29^c^  (-5.06) | 262.94± 19.86^bc^ (+8.63) | 555.77± 27.27^a^ (129.62) | 271.84 ±20.42^bc^  (+12.31) | 262.37 ± 54.25^bc^  (+ 8.40) | 309.70 ± 64.32^b^  (+27.95) |
| **SDH** | 74.94 ± 12.82^c^ - | 75.64 ± 13.35^c^ (+0.93) | 76.50 ± 9.93^c^  (+2.08) | 290.14 ±64.63^a^ (+287.16) | 71.03 ± 36.11^c^ (5.21) | 69.92 ± 35.93^c^  (-6.69) | 54.54 ± 3.64^c^  (-27.22) |
| **LDH** | 54.61 ± 4.21^b^ - | 57.51 ± 3.52^b^ (+4.65) | 59.51 ± 6.41^b^ (+8.97) | 163.58 ±15.85^a^  (+199.50) | 60.55 ± 11.42^bc^ (+10.87) | 67.19 ± 7.92^b^ (+23.03) | 64.26 ± 5.61^bc^  (+17.60) |
| **5^\^NT** | 52.12 ± 5.30^b^  - | 51.65 ± 5.24^b^ (-0.90) | 47.39 ± 7.55^b^  (-9.07) | 147.79 ±18.80^a^ (+183.55) | 51.17 ± 5.73^b^ (+13.98) | 56.45 ± 8.08^b^ (+8.30) | 55.22 ± 11.29^b^ (+5.94) |
| **AP** | 73.32 ± 5.96^b^ - | 73.92 ± 7.60^b^ (+0.81) | 72.74 ± 8.24^b^ (-0.79) | 270.75 ±98.09^a^ (+269.27) | 84.43 ± 5.25^b^  (-68.81) | 80.22 ± 8.16^b^ (+9.41) | 69.06 ± 7.65^b^ (-5.81) |

- Data are mean ± SD of eight rats in each group.
- Values are expressed as µmol/mg protein in all organelles marker enzymes.
- Statistical analysis is carried out by one way analysis of variance (ANOVA), Co-stat Computer Program accompanied by lease significance difference (LSD) test between groups at p<0.05.
- Shared letters are non-significant values between groups.
- Unshared letters are significant values between groups at p<0.0001.
- Values between brackets are percentage changes over control group.


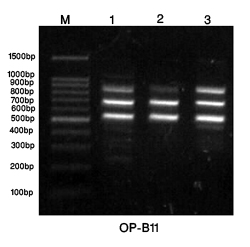

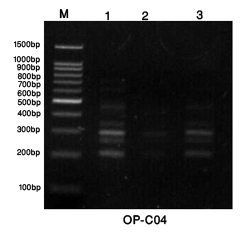

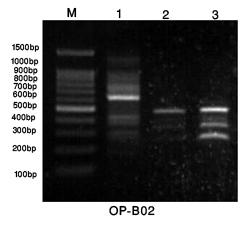

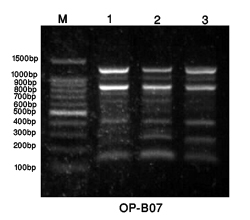

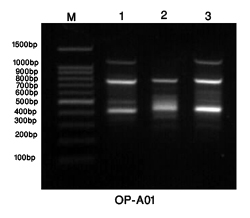


**A**


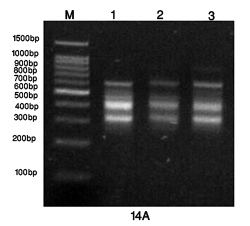

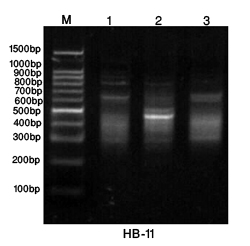

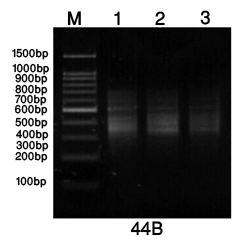

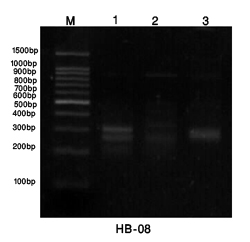

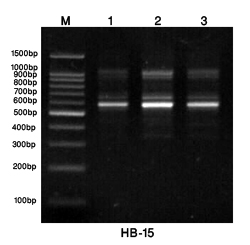


**B**

**Fig. *S*1.** A) The RAPD amplification electrophoretic profile of the investigated samples of *Annona squamosa, Annona cherimola* and *Annona Abdel Razek*. B) The ISSR amplification electrophoretic profile of the investigated samples of *Annona squamosa, Annona cherimola* and *Annona Abdel Razek* (the hybrid)

M= DNA marker (1 Kb ladder)

1= *Annona squamosa*, 2= *Annona cherimola* and 3= *Annona Abdel Razek*


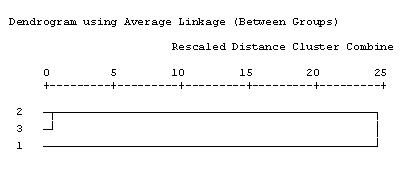


**Fig. *S*2.** The combination between the RAPD and ISSR data

Leaves

Barks

**B**

Barks

Leaves

**A**

**Fig *S*3:** A) Negative Mode & B) Positive Mode, UHPLC-MS traces of leaves and bark *Annona* Abdel Razek extracts showing different qualitative differences according to their metabolic profiles .

Dellaporta, S. L., J. Wood, J. B. Hicks (1983). A plant DNA minipreparation: version II. Plant molecular biology reporter 1, 19-21

Williams, J. G., A. R. Kubelik, K. J. Livak, J. A. Rafalski, S. V. Tingey (1990). DNA polymorphisms amplified by arbitrary primers are useful as genetic markers. Nucleic acids research 18, 6531-6535
